# Identifying the genes involved in the egg-carrying ovigerous hair development of the female blue crab *Callinectes sapidus*: transcriptomic and genomic expression analyses

**DOI:** 10.1186/s12864-023-09862-9

**Published:** 2023-12-11

**Authors:** Tao Wang, Tsvetan Bachvaroff, J. Sook Chung

**Affiliations:** 1 Department of Marine Biotechnology & Institute of Marine and Environmental Technology, University of Maryland Baltimore County, Baltimore, MD 21202 USA; 2 Institute of Marine and Environmental Technology, University of Maryland Center for Environmental Science, Baltimore, MD 21202 USA

**Keywords:** Female-specific characteristics, Crustacean female sex hormone, Ovigerous hairs, Female blue crabs, Genome, Transcriptome

## Abstract

**Background:**

Crustacean female sex hormone (CFSH) controls gradually developing adult female-specific morphological features essential for mating and brood care. Specifically, ovigerous hairs are developed during the prepuberty molt cycle of the blue crab *Callinectes sapidus* that are essential for carrying the eggs until they finish development. Reduced *CFSH* transcripts by *CFSH*-dsRNA injections result in fewer and shorter ovigerous hairs than the control. This study aimed to identify the specific genes responsible for ovigerous hair formation using transcriptomic, genomic and expression analyses of the ovigerous setae at three stages: prepuberty at early (OE) and late premolt (OL), and adult (AO) stages.

**Results:**

The *de novo* Trinity assembly on filtered sequence reads produced 96,684 Trinity genes and 124,128 transcripts with an N50 of 1,615 bp. About 27.3% of the assembled Trinity genes are annotated to the public protein sequence databases (i.e., NR, Swiss-Prot, COG, KEGG, and GO databases). The OE vs. OL, OL vs. AO, and OE vs. AO comparisons resulted in 6,547, 7,793, and 7,481 differentially expressed genes, respectively, at a log2-fold difference. Specifically, the genes involved in the Wnt signaling and cell cycle pathways are positively associated with ovigerous hair development. Moreover, the transcripts of ten cuticle protein genes containing chitin-binding domains are most significantly changed by transcriptomic analysis and RT-qPCR assays, which shows a molt-stage specific, down-up-down mode across the OE-OL-AO stages. Furthermore, the expression of the cuticle genes with the chitin-binding domain, Rebers and Riddiford domain (RR)-1 appears at early premolt, followed by RR-2 at late premolt stage. Mapping these 10 cuticle protein sequences to the *C. sapidus* genome reveals that two scaffolds with a 549.5Kb region and 35 with a 1.19 Mb region harbor 21 RR1 and 20 RR2 cuticle protein genes, respectively. With these findings, a putative mode of CFSH action in decapod crustaceans is proposed.

**Conclusions:**

The present study describes a first step in understanding the mechanism underlying ovigerous hair formation in *C. sapidus* at the molecular level. Overall, demonstrating the first transcriptome analysis of crustacean ovigerous setae, our results may facilitate future studies into the decapod female reproduction belonging to the suborder Pleocyemata.

**Supplementary Information:**

The online version contains supplementary material available at 10.1186/s12864-023-09862-9.

## Background

Decapod crustaceans, especially economically important species, are sexually dimorphic, and their reproductive strategy and behavior involve diverse adult stage-dependent female characteristics. The features related to adult specific-sexual maturity are more evident in egg carriers of Pleocyemata (crayfish, lobsters, prawns, and crabs) [1] than in egg releasers of Dendrobranchiata (shrimp). The former retains fertilized eggs on the ovigerous setae borne on the distal segment of maternal pleopods located inside or beneath the abdomen or under the tail until hatching [2]. In contrast, the extruded eggs of the latter are released directly into the water [3].

In the blue crab, *Callinectes sapidus*, adult females attach 0.7-6 million embryos only to the ovigerous setae using the embryo attachment system [4, 5] that allows for maternal care of the embryogenesis during brooding [6]. Additionally, the female *C. sapidus* abdominal shape transforms from triangular to semicircular after the puberty-terminal molt. This change increases the surface area to attach more embryos to the ovigerous setae [4].

The first identification of crustacean female sex hormone (CFSH), a novel protein hormone from the eyestalk ganglia of *C. sapidus* [7], has led to its discovery in other decapod crustacean species [8–11]. This hormone develops adult-female-specific phenotypes, including abdominal shape, gonopores, and ovigerous hairs on ovigerous setae [7, 12]. Interestingly, reduced *CFSH* levels by *CFSH*-double stranded RNA (dsRNA) injection into prepuberty females at specific molt stages results in pronounced phenotype changes in mainly abdominal size and cuticular structured-ovigerous hair length and abundance [13]. These findings suggest that the constant presence of CFSH may play pivotal roles in gradually developing adult-female-specific morphological features, possibly via cuticle gene expressions during prepuberty molt stages.

The exact mechanism of CFSH in regulating the development of these adult-female-specific characteristics has yet to be elucidated. However, high levels of 17β-estradiol (E2) are present in the ovigerous setae producing ovigerous hairs in prepuberty females [14]. Moreover, long-term *CFSH*-dsRNA administration reduces E2 levels and transcripts of steroidogenesis-related genes in adult-female-specific tissues [14]. These findings indicate that CFSH may involve E2 on the target tissue, ovigerous setae, for ovigerous hair formation.

The next-generation sequencing (NGS) has been employed for the functional genomics of non-model organisms. For example, transcriptome datasets of the following crustacean tissues include the androgenic gland [15, 16], gonads [17, 18], Y-organ [19–21], hemolymph [22], and limb bud [23] are obtained for studies on reproduction, molting, immunity, regeneration, and other biological processes. In this study, the RNA sequencing (RNA-seq) with the blue crab genome available [24] was employed to determine the putative genes involved in the ovigerous hair formation that are most critically affected by *CFSH*-dsRNA injections [7, 13].

Herein, we present a comparative analysis of the transcriptome data derived from the ovigerous setae of *C. sapidus* in prepuberty (early and late premolt) and adult females using RNA-seq. An annotated ovigerous setae transcriptome was constructed via *de novo* assembly of sequences and mapped in the blue crab genome. Furthermore, cuticle and tubulin genes were the most abundant transcripts in the prepuberty females at the late premolt dataset, confirmed by RT-qPCR assays. The genes involved in the Wnt signaling and cell cycle were positively associated with ovigerous hair development. Finally, these findings were incorporated into a model describing a putative mode of CFSH action in developing ovigerous hair of decapod crustaceans.

## Methods

### Animal culture and tissue collection

Adult (135.4 ± 5.3 mm carapace width (CW), *n* = 5) and prepuberty (95.3 ± 3.7 mm CW, *n* = 10) females of *C. sapidus* were produced in the blue crab hatchery, Aquaculture Research Center, Institute of Marine and Environmental Technology, Baltimore, MD, USA [13]. Prepuberty females were molt staged as follows: early premolt (D_0_, *n* = 5) and late premolt (D_2 − 3_, *n* = 5) [25]. The animals were reared in individual compartments (15 cm × 15 cm) in a closed recirculating aquaculture system with 25 ppt artificial seawater at 22–23 °C [26]. The water quality was monitored daily by ZooQuatic Lab (Baltimore, MD). Animals were cultivated until they reached the target molt stage by feeding daily with a piece of frozen squid (10% of body weight) [26] between 8 and 10 A.M.

All animals were sacrificed between 2 and 6 P.M. (summer 2019) after placing them on ice for 10 min before dissection. The ovigerous setae (prepuberty females at early: OE and late premolt: OL were cut and dissected in ice-cold diethylpyrocarbonate (DEPC)-treated crustacean saline under a stereomicroscope (Leica), placed on dry ice immediately, and stored at -80˚C until analysis. After completing embryogenesis and releasing the larvae, the adult female ovigerous setae: AO were dissected similarly. The molt stage of these animals was at the intermolt stage [25].

### RNA extraction, library preparation, and sequencing

Total RNAs from the ovigerous setae were extracted using QIAzol Reagent (Qiagen, Germany) following the manufacturer’s procedures. RNA concentrations were measured using a Nanodrop (Thermo Scientific, US). Equal amounts of total RNA from 3 to 5 individuals at the same stage were pooled for sequencing (www.macrogenusa.com). The quantity and quality of each RNA sample were checked using a Bioanalyzer (Agilent, US).

### De novo assembly and annotation

The quality of paired-end 150 base sequencing of Illumina raw reads was evaluated using FastQC. These Illumina raw reads were pre-processed to remove reads containing adapter and poly-N and low-quality reads using Trimmomatic v0.35 with the following default parameters (SLIDINGWINDOW:4:5 LEADING:5 TRAILING:5 MINLEN:25) [27]. Clean reads were assembled as a reference transcriptome using Trinity v2.14.0 with default parameters (read normalization maximum 200, minimum contig size 200, kmer size 25) [28, 29]. Following assembly, the contigs were clustered based on a 95% sequence similarity threshold using CD-HIT-EST v4.6.6 [30]. The completeness of the clustered and unclustered assemblies was evaluated using Benchmarking Universal Single-Copy Orthologs (BUSCO) v5.3.2 analysis [31]. The analysis was performed by comparing it against the arthropoda_odb10 lineage dataset (1013 genes) and using the dependencies augustus-3.3.1 and hmmer-3.2.1 with a default e-value threshold of 1e-05.

Trimmed reads were mapped back to the clustered assembly using Bowtie2 v2.1.0. Homology search was conducted using BLASTx against the NCBI non-redundant protein (NR), Swiss-Prot (protein sequences), Clusters of Orthologous Groups (COG), Kyoto Encyclopedia of Genes and Genomes (KEGG), and Gene Ontology (GO) databases with a default cut-off e-value of 1e-05. Gene functional annotations were obtained according to the best alignment results.

### Differentially expressed genes (DEGs) and enrichment analysis

Relative gene expression was calculated and normalized to the transcripts per million (TPM) using RSEM v1.2.28 [32]. DEGs were identified using edgeR v3.34.0 with default criteria: *p-*value *<* 0.05 and |log2-fold change| *>* 1. Unique and shared transcripts were visualized using the UpSet plot (https://gehlenborglab.shinyapps.io/upsetr/) [33]. DEGs were then subjected to enrichment analysis of GO functions and KEGG pathways [34].

Enrichment analysis of DEGs was conducted using ClusterProfiler v4.0.2 R package. The DEGs were considered significantly enriched in a particular KEGG Orthology (KO) or KEGG category at *p-*value < 0.05. The KEGG Automatic Annotation Server (http://www.genome.jp/tools/kaas/) was used to obtain KO numbers to summarize the KO terms associated with annotated transcripts in ovigerous setae transcriptome assembly. The Venn diagram and the heat map were constructed using ImageGP (http://www.ehbio.com/ImageGP) [35].

### Sequence and phylogenetic analyses

The nucleotide and amino acid sequences of selected genes were examined using BLAST (http://blast.ncbi.nlm.nih.gov/Blast.cgi), with an e-value threshold of 1e-06. The open reading frames (ORFs) were identified using ORFfinder (www.ncbi.nlm.nih.gov/orffinder/). BLASTP further validated the predicted ORFs against the NR database. A 2-kb upstream promoter of genes was extracted from the blue crab reference genome [24] and was predicted using BDGP (https://www.fruitfly.org/seq_tools/promoter.html). The potential transcriptional factor binding sites were analyzed using AliBaba 2.1 (http://gene-regulation.com).

Phylogenetic analysis was conducted using the amino acid sequence of genes from several representative insects and crustaceans retrieved from the NCBI database (Additional Table 1). A phylogenetic tree was constructed based on the deduced full-length amino acid sequence alignments by the Neighbor-Joining (NJ) algorithm embedded method in the MEGA X and modified by the iTOL (https://itol.embl.de/).

### Expression based on RNA-seq mapping to the genome

As further validation of the *de novo* assembled transcriptome, read mapping against the genome was also used to infer transcript sequences and abundance. First the reads were mapped against the reference genome (JAHFWG010000000) using hisat2 (https://registry.opendata.aws/jhu-indexes/), followed by StringTie (https://ccb.jhu.edu/software/stringtie/) transcriptome prediction of the transcriptome and estimation of relative abundance TPM values. These predicted genes were then used to search against the genome, predicted transcriptome, and *de novo* assembly with BLASTn. For comparisons with the *Portunus trituberculatus* genome, BLASTx was used against the NCBI database.

### Expression analysis of selected genes using RT-qPCR

To confirm the relative abundance TPM values of the selected genes, the ovigerous setae were collected from the early and premolt stage of the prepuberty females and spawned females described in Sect. 2.1. The expression levels of selected genes were validated using RT-qPCR assays. The total RNA samples (1.5 µg) were reverse transcribed using a PrimeScript^RT^ with gDNA eraser (TaKaRa) and assayed using a Fast SYBR Green Master Mix (Applied Biosystems, US) and gene-specific primers (Additional Table 2). The dissociation curve analysis was added to the RT-qPCR assays to verify the amplicon sizes, and the standards were prepared following the procedures described [36]. The cDNA samples (containing 25 ng total RNA) and standards were assayed in duplicate. The data were analyzed as described [36]. In brief, the data were normalized against the reference genes, arginine kinase (*AK*) and eukaryotic translation initiation factor 4 A (*eIF4A*) expression levels in the same cDNA samples and presented as mean ± SE (*n* = 4–5) transcripts/µg total RNA.

### Statistical analysis

Statistical significances of the expression data among different stages were determined using SPSS 25.0 program, with one-way ANOVA followed by Dunn’s posthoc test. Different lowercase letters show significance at *p* ˂ 0.05.

## Results

### Sequencing and read assembly

A total number of ~ 37, 38, and 48 million raw reads were obtained from RNA from the OE, OL, and AO samples (Table 1). About 90.6, 92.0, and 91.9% of reads were retained after pre-processing to discard low-quality reads. A total of 153,813,359 base pairs (bp) were assembled, generating 99,046 Trinity genes and 158,749 Trinity transcripts. The average GC content was 44.3%, and N50 was 2,001 bps long.

**Table 1:** Summary of the *de novo* assembly statistics of *C. sapidus* ovigerous setae transcriptome and mapping. Transcriptome: prepuberty females at early (OE) and late premolt (OL), and adult stages (AO). *De novo* assembly statistics are based on all contigs

Applied software	Assembly statistics	Trinity		Trinity95	
FastQC (evalulation) & Trimmomatic (processing)	Total assembled bp	153,813,359		102,627,517	
Trinity & CD-HIT-EST	Number Trinity genes	99,046		96,684	
Trinity & CD-HIT-EST	Number Trinity transcripts	158,749		124,128	
	Average contig (bp)	968.9		826.8	
	GC (%)	44.3		44.2	
	N50	2,001		1,615	
	**Mapping**	**OE**	**OL**		**AO**
	Total raw reads	37,084,746	37,793,011		48,109,102
FastQC (evalulation) & Trimmomatic (processing)	Total clean reads	33,584,743	34,742,059		44,216,393
Bowtie2	% reads mapped to Trinity95	96.1%	92.1%		91.5%
Bowtie2	Total number of contigs	56,432	91,353		99,493
	Number of filtered contigs (TPM ≥ 1)	18,867	31,605		32,021
	Average filtered contig (bp)	1,191.4	1,321.0		1,425.9

**Fig. 1:**
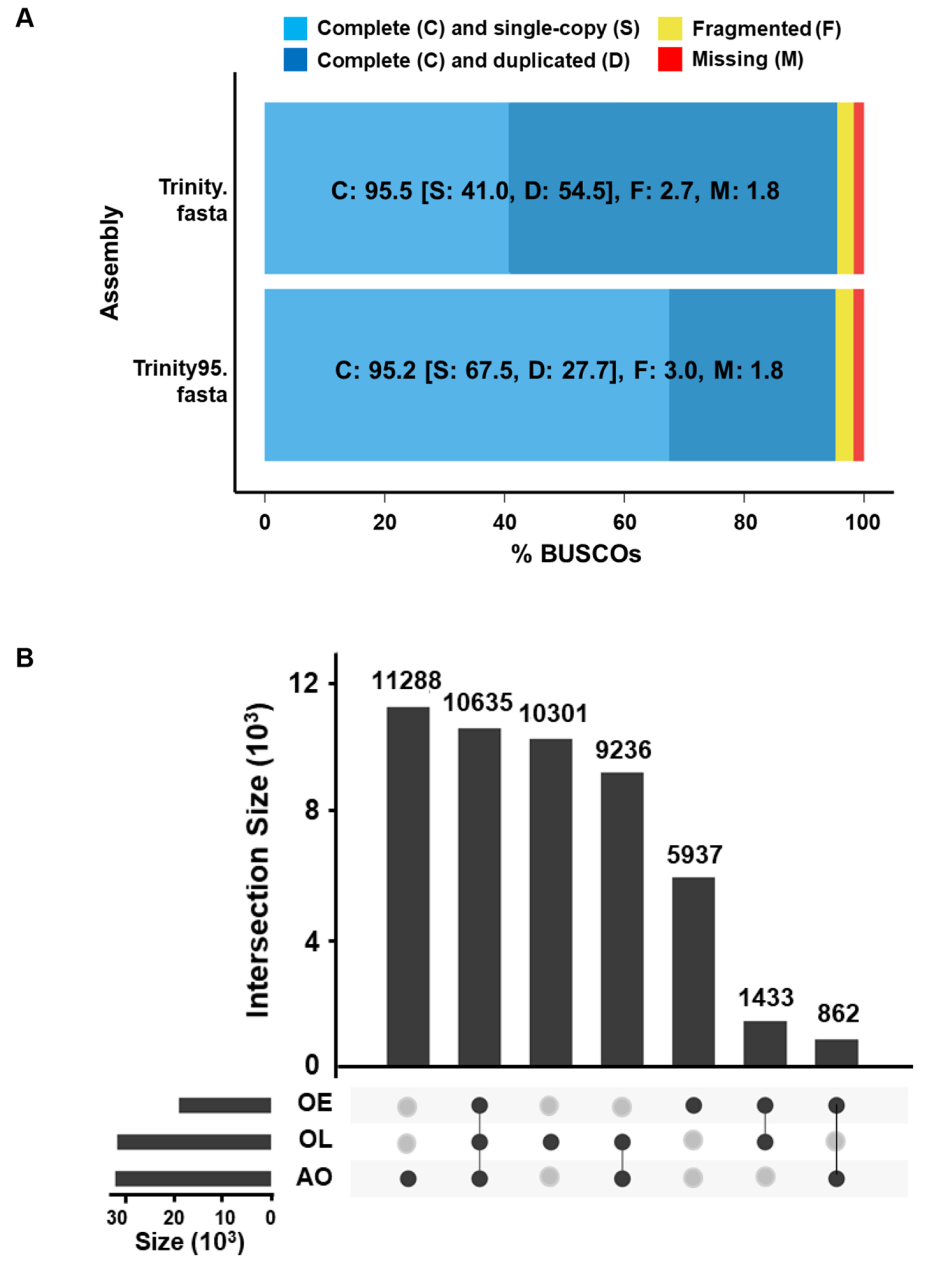
**(A)** BUSCO assessment results of the Trinity and Trinity_95 transcriptome against the Arthropod reference; **(B)** UpSet plot depicting the number of unique and shared transcripts with TPM ≥ 1 in each dataset. Abbreviation: ovigerous setae in the prepuberty females at early (OE) and late premolt (OL) and adult (AO) stages

The redundancy of the Trinity assembly was then filtered using CD-HIT-EST, resulting in 96,684 Trinity genes (97.6% retained) and 124,128 Trinity transcripts (78.2% retained) in the Trinity95 clustered dataset (Table 1). The BUSCO analysis revealed that the annotated genes and transcripts were highly complete and covered more than 95% of BUSCOs in Trinity and Trinity95 assemblies (Fig. 1A). The resulting Trinity95 assembly increased single-copy genes from 41.0 to 67.5% and duplicates decreased from 54.5 to 27.7% compared to the Trinity assembly, suggesting that the Trinity95 assembly was complete. Therefore, the data were used as the reference transcriptome for further mapping analysis.

Read alignment was similar among the three datasets, ranging from 91.5 to 96.1% of the reads mapped back to the Trinity95 assembly. After filtering with a TPM cut-off of 1, the AO dataset had the most transcripts with 32,021 sequences, followed by the OL with 31,605 and 18,867 in the OE. The UpSet plot showed the intersection between the three transcriptomes (Fig. 1B). OE, OL, and AO datasets possessed 5,937, 10,301, and 11,288 unique transcripts, respectively, while sharing 10,635 transcripts.

### Functional annotation

**Fig. 2:**
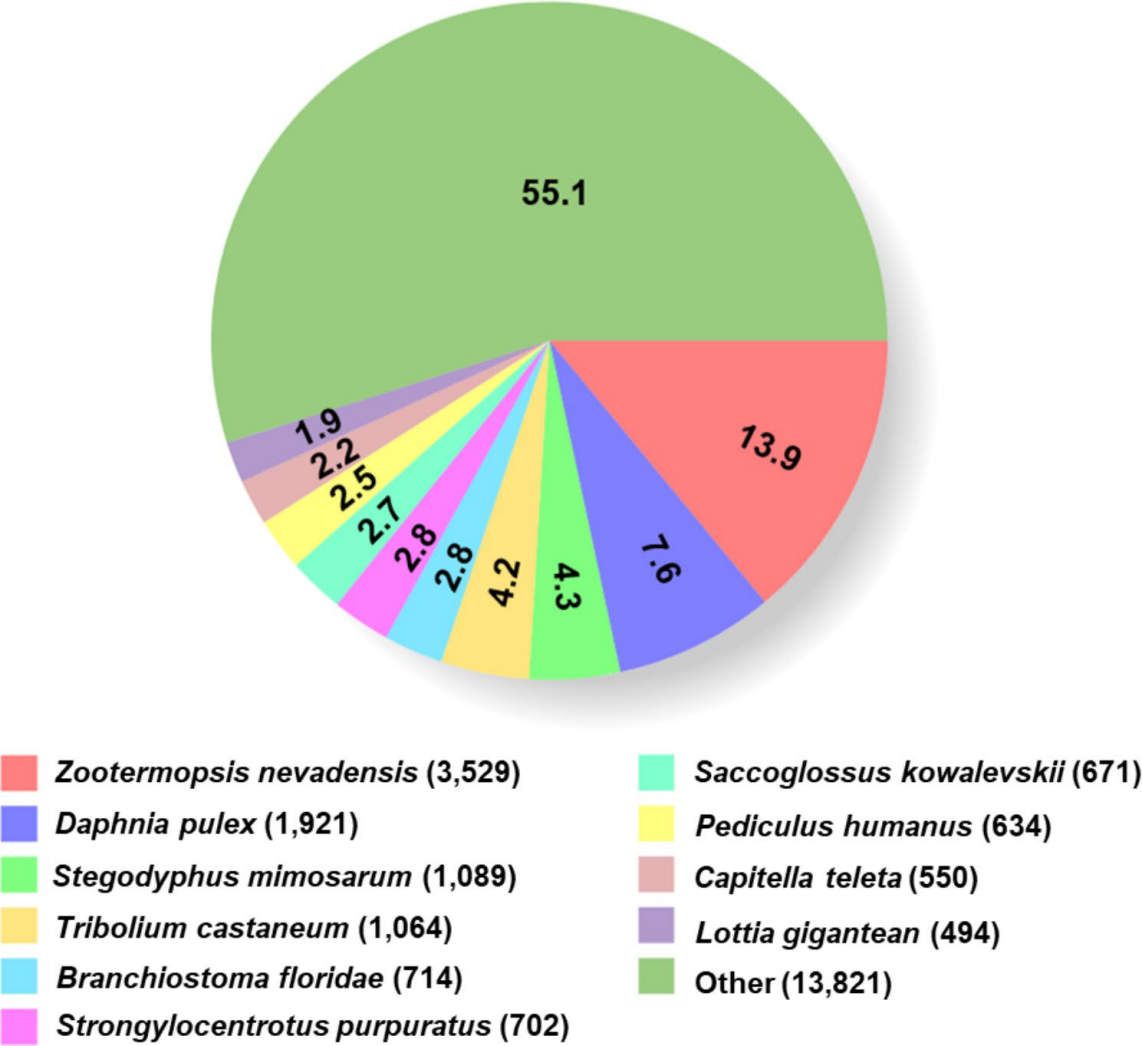
Functional classification of *de novo* assembled *C. sapidus* ovigerous setae transcriptome. Species distribution shown as a percentage of total homologous sequences with E-value ≥ 1e-05

Of 96,684 Trinity genes, 25,215 (26.1%) were matched to the NR database. In comparison, only 6,796 (7.0%) genes showed significant matches in the COG database (Table 2). The species showing the top five BLASTx hits were *Zootermopsis nevadensis* (13.9%), followed by *Daphnia pulex* (7.6%), *Stegodyphus mimosarum* (4.3%), *Tribolium castaneum* (4.2%), and *Branchiostoma floridae* (2.8%). However, “other” species was the largest group (13,894 unigenes; 55.1%) (Fig. 2).

**Table 2:** Summary of functional annotation of *C. sapidus* transcriptomes

Databases	Unigene number	Percentage (%)
NR	25,215	26.1
Swiss-Prot	13,932	14.4
COG	6,796	7.0
KEGG	16,309	16.9
GO	13,772	14.3
Total	26,362	27.3

### Enriched pathways in differentially expressed genes

**Fig. 3:**
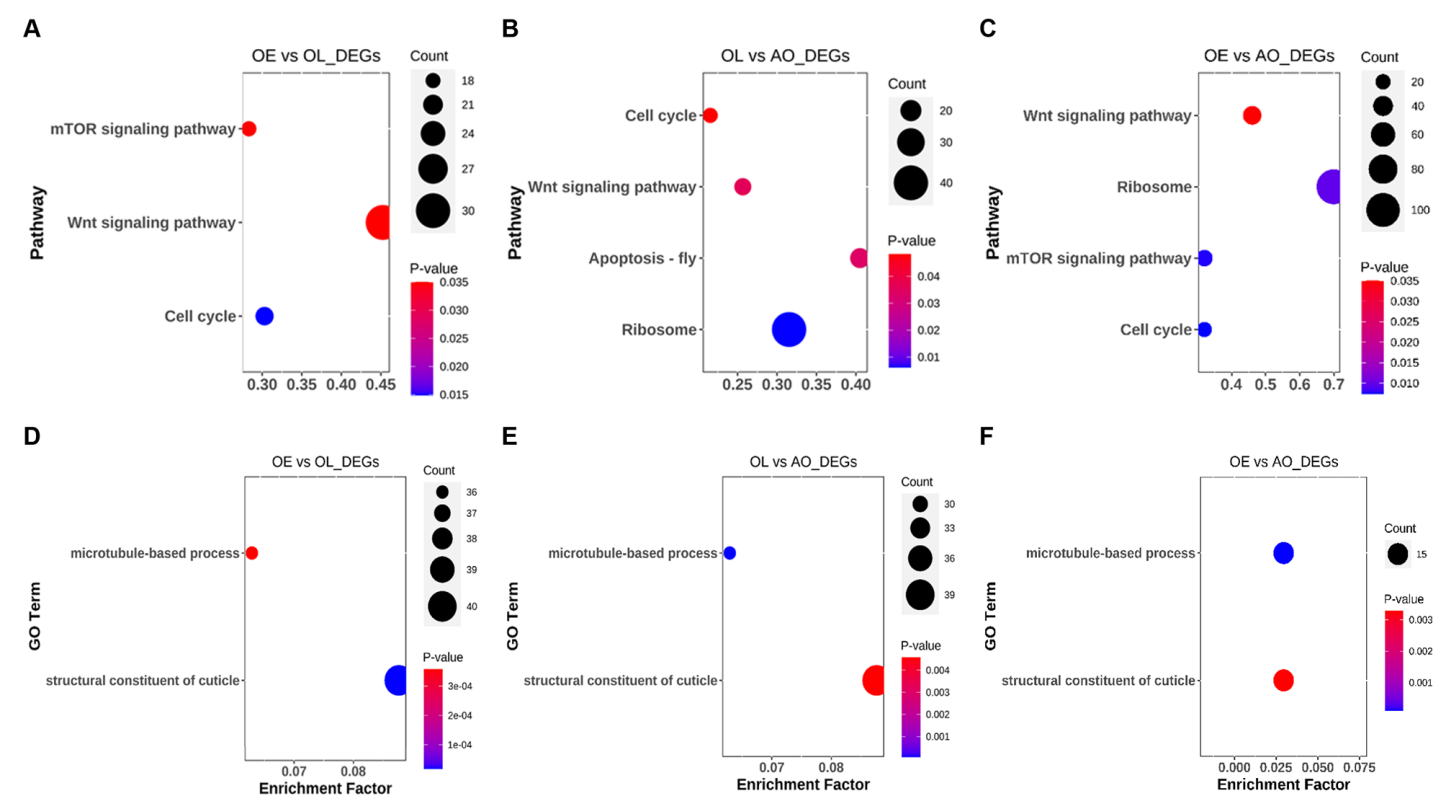
Enrichment analyses of differentially expressed genes (DEGs). Kyoto Encyclopedia of Genes and Genomes (KEGG) enrichment analysis of DEGs in the **(A)** OE vs. OL, **(B)** OL vs. AO, and **(C)** OE vs. AO comparisons, respectively. The top-enriched Gene Ontology (GO) terms derived from the GO enrichment analysis of DEGs in the **(D)** OE vs. OL, **(E)** OL vs. AO, and **(F)** OE vs. AO comparisons, respectively. “Count” represents the number of DEGs enriched in certain pathways. “*p*-value” is the value obtained by hypergeometric test to define the significance of enriched pathways, and its colors represents the significance from blue (low) to red (high). The arrows indicate key pathways. Abbreviation: ovigerous setae in the prepuberty females at early (OE) and late premolt (OL) and adult (AO) stages The Kanehisa laboratory have kindly provided permission [34] (www.kegg.jp/kegg/kegg1.html)

A total of 6,547, 7,793, and 7,481 DEGs were identified in the OE vs. OL, OL vs. AO, and OE vs. AO comparisons (Additional file 1). The putative functions of DEGs were assigned using KEGG and GO pathway enrichment analyses (Additional file 2). Three signaling pathways were enriched by annotated DEGs, including the mechanistic target of rapamycin (mTOR) signaling pathway (ko04150), wingless-type MMTV integration site family (Wnt) signaling pathway (ko04310), and cell cycle pathway (ko04110) (Fig. 3A-C). The “Apoptosis-fly (ko04214)” pathway was uniquely enriched in the OL vs. AO comparison, while most DEGs were significantly enriched in the “ribosome” (ko03010) in the OL vs. AO and OE vs. AO comparisons. On the other hand, the most significantly over-represented GO terms in three comparisons were microtubule-based process (GO: 0007017)” in the Biological Process category and “structure constituent of the cuticle (GO: 0042302)” in the Molecular Function category (Fig. 3D-F).

### Transcripts involved in the wnt signaling and cell cycle pathways and expression analysis

**Fig. 4:**
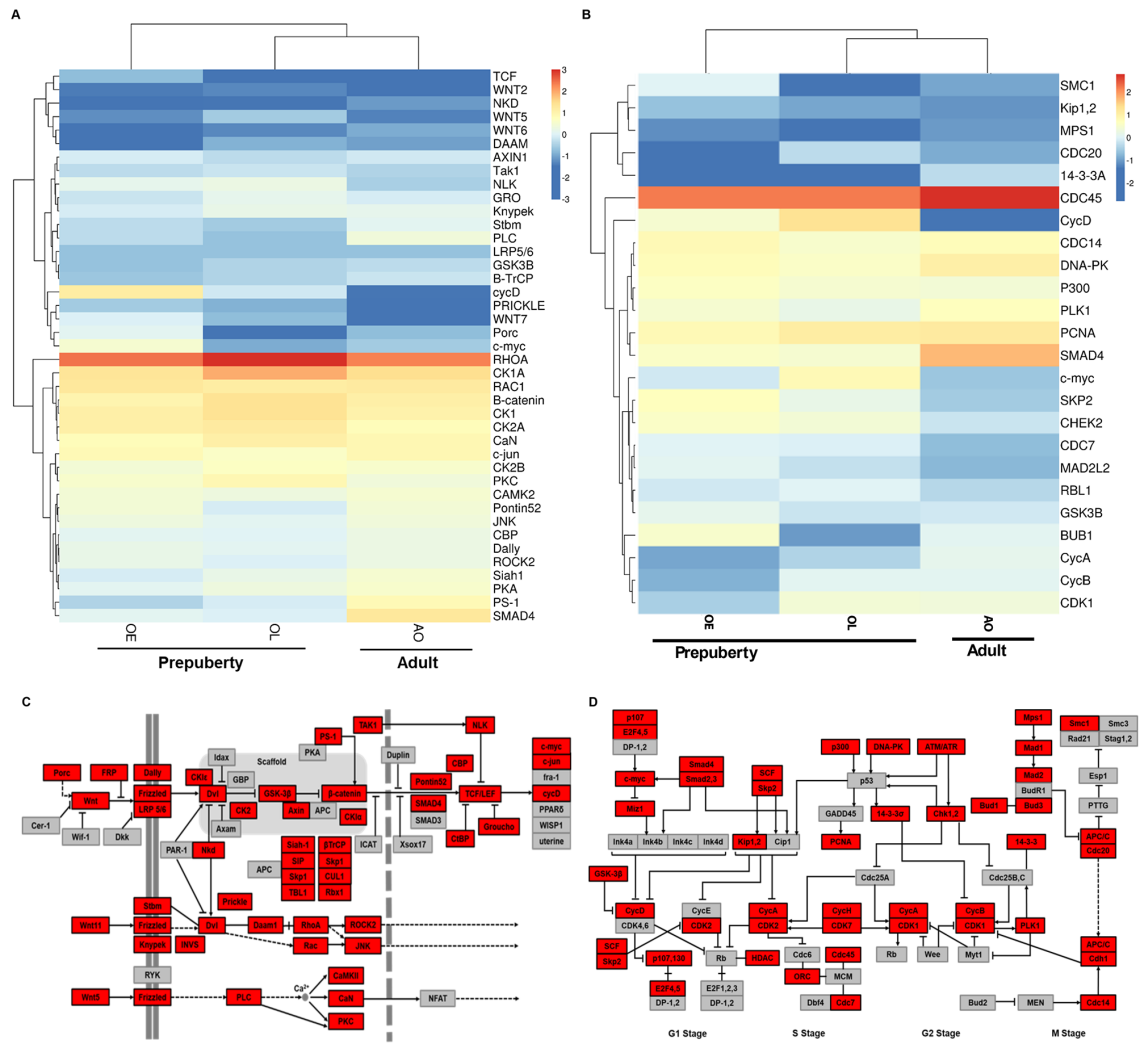
The heat map showing the log2-fold change of the TPM values of DEGs involved in the Wnt signaling **(A)** and cell cycle **(B)** pathways, respectively. The Wnt signaling **(C)** and cell cycle **(D)** pathways generated by KEGG pathway analysis representing the present and absent gene products in the transcriptome of *C. sapidus*. Gene products appearing in the *C. sapidus* transcriptome are represented in red-colored boxes. Pathway schematics modified from KEGG pathway model images (www.kegg.jp/kegg/pathway.html). Abbreviation: ovigerous setae in the prepuberty females at early (OE) and late premolt (OL) and adult (AO) stages. The Kanehisa laboratory have kindly provided permission [34] (www.kegg.jp/kegg/kegg1.html)

The components of the Wnt signaling and cell cycle were represented in the transcriptome (Fig. 4). Phylogenetic analysis was performed to validate the wnt signaling and cell cycle genes. The putative amino acid sequences of crustacean *Wnt5b*, *β-catenin*, *cyclin A*, *cyclin D2-like*, *cyclin H*, and *CDC20* were aligned with representative arthropods and in phylogenies were grouped and distinct from insect outgroup sequences (Additional file 3 A-F). DEG analysis revealed increased transcript levels for the following critical Wnt signaling pathway genes from OE to OL: *Wnt2*, *Wnt5*, *Wnt6*, *low-density lipoprotein receptor-related protein* (*LRP*), *Frizzled*, *casein kinase 1* (*CK1*), *CK2*, *β-catenin*, *glycogen synthase kinase-3β* (*GSK3β*), *Axin1*, and *groucho* (*GRO*) (Fig. 4A&C; Additional Table 3). However, transcript levels for these genes did not differ between OL and AO.

**Table 3:** Abundance of genes in transcript per million (TPM) involved in structural constituent of cuticle and microtubule-based process in the transcriptomes of *C. sapidus* ovigerous setae. Transcriptomes: prepuberty females at early premolt (OE) and late premolt (OL), and adults (AO). |log2 (fold change)| < 1 is noted by ‘-’ symbols. Expression was also determined using genome mapping of the RNAseq data in the last three columns

**Seq ID**	Gene Name	Gene Abbreviation		*C. sapidus* Genome Location		Top NCBI Accession NO.	Trinity TPM Value				Trinity Log2 (fold change) Value				Genome mapping TPM value		
							OE		OL	AO	OE vs. OL	OL vs. AO		OE vs. AO	OE	OL	AO
**Structural constituent of cuticle: chitin_bind_4 domain with RR-1 sequence**																	
TRINITY_DN9_c0_g1	arthrodial membrane protein AMP1A-like	AMP1A-like			HiC_scaffold_20: 23834608.. 24379310	XP_045136044.1	2.24	4,372.78		2.17	12.73	-9.33		-	0	3,497.74	0.17
TRINITY_DN333_c4_g1	arthrodial membrane protein AMP8.1	AMP8.1			HiC_scaffold_16: 5226063..5227150	AAV28476.1	6.97	1,506.03		301.52	6.40	-2.70		5.43	4.85	1,071.43	312.86
TRINITY_DN44_c1_g2	larval cuticle protein LCP17-like	LCP17-like			HiC_scaffold_16: 3902805..3903928	XP_045101992.1	11.09	525.71		22.22	4.23	-4.94		-	8.52	450.78	29.98
TRINITY_DN86_c2_g1	calcified cuticle protein CP8.5	CP8.5			HiC_scaffold_26: 11503353..11504081	AAV28478.1	0.14	238.01		0.08	9.37	-6.16		-	0	245.13	0
**Chitin_bind_4 domain with RR-2 sequence**																	
TRINITY_DN11_c0_g1	cuticle protein 7-like	CP7-like			HiC_scaffold_35: 1259650..2443401	XP_045107367.1	1.37	14,684.52		0.74	12.50	-15.12		-	0.88	15,067.56	1.06
TRINITY_DN1246_c1_g1	adult-specific cuticle protein ACP20	ACP20			HiC_scaffold_11024: 1191..1640	XP_045101342.1	0.47	3,109.80		1.53	11.35	-5.72		1.70	0.57	4,269.25	0.67
TRINITY_DN38_c23_g2	pro-resilin	Pro-resilin			HiC_scaffold_27: 4189445..4189982	XP_042211318.1	0.12	991.54		0.04	11.64	-14.94		-1.58	0.27	1149.54	0.068
TRINITY_DN2304_c0_g1	cuticle protein 6	CP6			HiC_scaffold_20: 24226901..24228133	MPC43157.1	0.12	455.13		0.40	10.31	-4.86		1.74	0.11	167.07	0.546
TRINITY_DN181_c0_g1	cuticle protein 8-like	CP8-like			HiC_scaffold_28: 10632703..10633852	XP_045101387.1	0.03	111.22		0.05	10.31	-5.68		-	0	97.30	0.06
**Chitin_bind_4 domain without RR sequence**																	
TRINITY_DN5111_c0_g1	calcified cuticle protein CP15.0	CP15.0			HiC_scaffold_37: 4750563..4751141	ABB91679.1	0.15	114.43		0.01	8.16	-8.88		-3.91	0	95.48	0
**Microtubule-based process**																	
TRINITY_DN19_c0_g1	tubulin alpha-1 chain		TUBA1	HiC_scaffold_15: 1506280..1511624		XP_045137688.1	1,214.19		2,233.57	780.17	-	-1.90	-				
TRINITY_DN197_c34_g1	tubulin beta-1 chain		TUBB1	HiC_scaffold_33: 21557045..21562537		XP_045129538.1	1,076.71		946.54	411.23	-	-1.10	-1.54				

Transcript abundance for cell cycle genes encoding cyclin B and cyclin-dependent kinase 1 (*CDK1*) decreased by ~ 50% from OE to OL and then increased by about 8-fold for AO (Fig. 4B&D; Additional Table 3). In contrast, a reversed expression pattern was observed for *CDK2*, *CDK7*, cell division control protein 20 (*CDC20*), *G1/S-specific cyclin D2-like* (*cyclin D2-like*), and *cyclin H*. For the genes encoding cyclin A and *CDC45*, transcript levels in the OL library were marginally increased relative to OE, and then increased in the AO library.

**Fig. 5:**
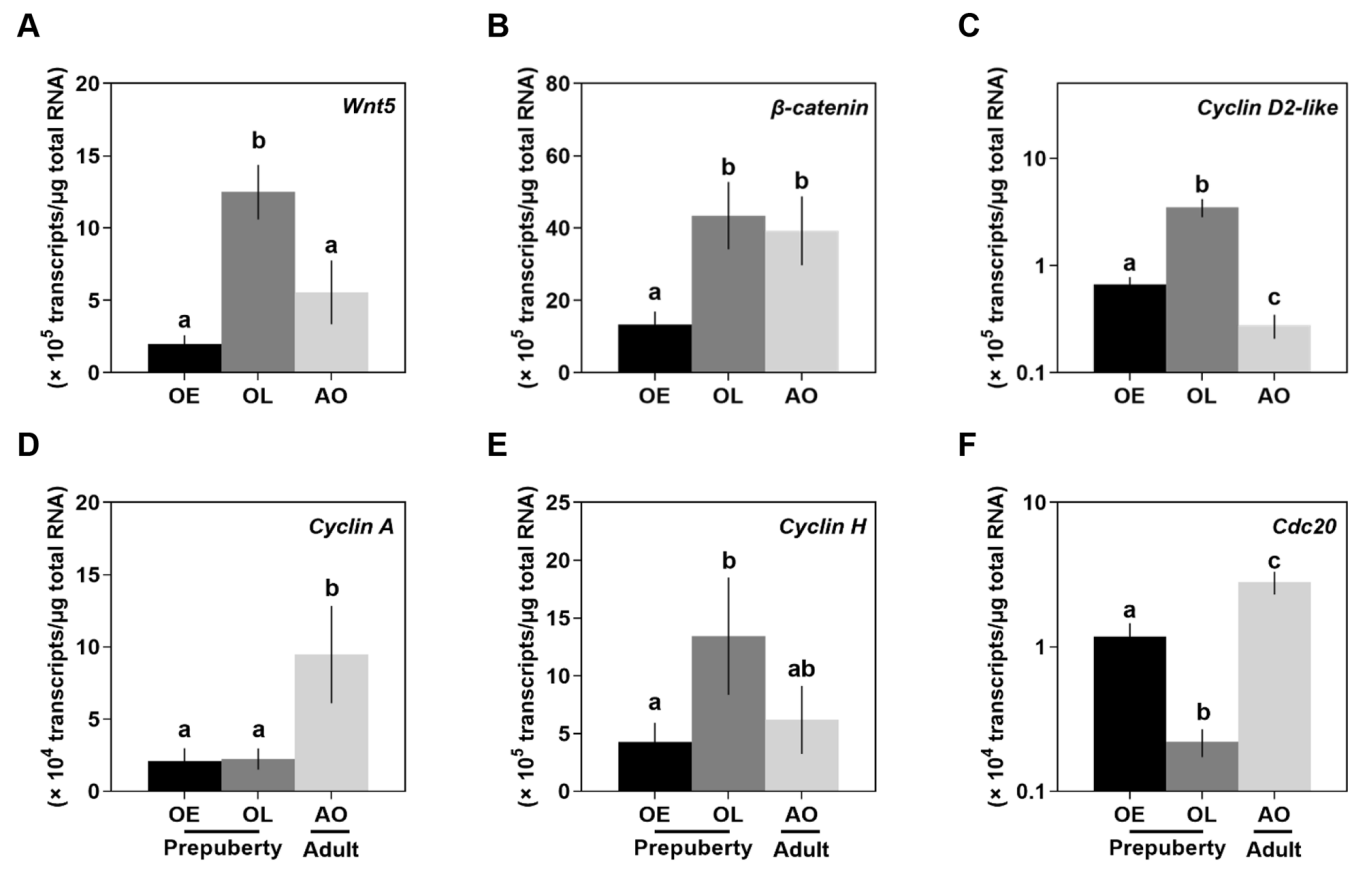
Expression profiles of DGEs involved in the Wnt signaling and cell cycle pathways in the ovigerous setae at different stages by RT-qPCR analysis (A-F). The data are represented as mean ± SE (*n* = 4–5) transcripts/µg total RNA. Statistical significance of the RT-qPCR data were determined using one-way ANOVA followed by Dunn’s posthoc test. Different lowercase letters show significance at *p* ˂ 0.05. Abbreviation: ovigerous setae in the prepuberty females at early (OE) and late premolt (OL) and adult (AO) stages

Based on DEGs data, *Wnt5*, *β-catenin*, *cyclin D2-like*, *cyclin A*, *cyclin H*, and *CDC20* levels were validated using RT-qPCR analyses (Fig. 5A-F). The prepuberty female ovigerous setae at late premolt had the highest *Wnt5* (12.5 ± 1.9 × 10^5^ transcripts/µg total RNA, *n* = 4), *β-catenin* (43.4 ± 9.3 × 10^5^ transcripts/µg total RNA, *n* = 4), *cyclin D2-like* (3.5 ± 0.7 × 10^5^ transcripts/µg total RNA, *n* = 4), and *cyclin H* (13.4 ± 5.1 × 10^5^ transcripts/µg total RNA, *n* = 4) transcripts (Fig. 5A-C & E), compared to the other two stages. Adult ovigerous setae had the most abundant transcript level of *cyclin A* (9.5 ± 3.4 × 10^4^ transcripts/µg total RNA, *n* = 4) and *CDC20* (2.8 ± 0.5 × 10^4^ transcripts/µg total RNA, *n* = 4) (Fig. 5D & F). Overall, these transcript levels measured using RT-qPCR agreed with the relative abundance TPM values.

### DEGs related to the structural constituent of cuticle and microtubule-based process

**Fig. 6:**
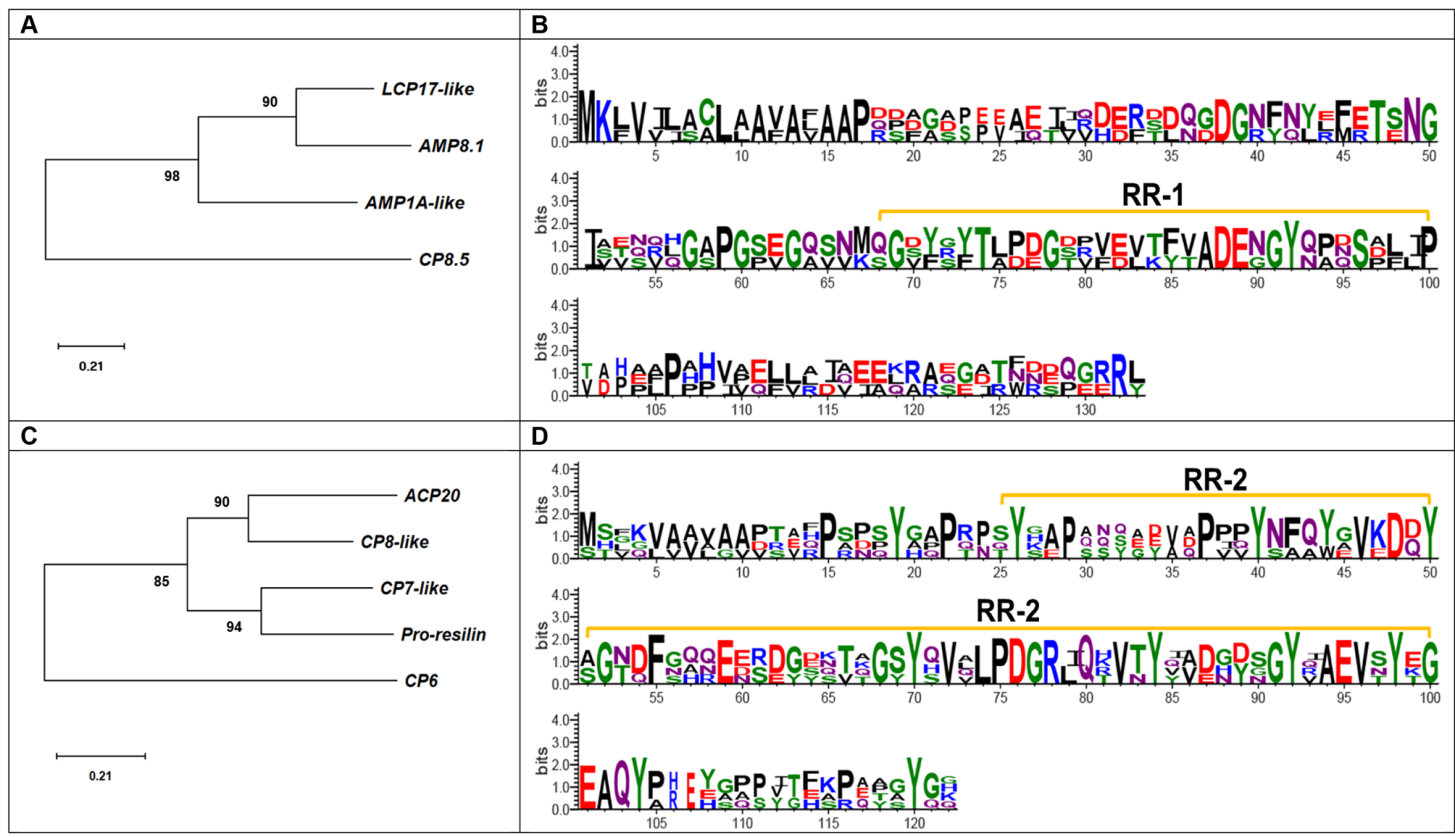
**(A)** Phylogenetic tree of *C. sapidus* cuticle proteins was constructed using neighbor-joining (NJ) approach in MEGA X. Bootstrap consisted of 1000 replicates (Fig. 6A-B). A sequence logo of MEGA X aligned AMP1A-like, AMP8.1, and LCP17-like **(B)** and CP7-like, CP8-like, ACP20, and pro-resilin **(D)** was generated using the WebLogo 3.0 (https://weblogo.threeplusone.com/create.cgi). The Y-axis describes the amount of information in bits*, amino acid size is proportional to frequency, and the X-axis shows the position in the alignment. The Rebers-Riddiford (RR) consensus sequence region (RR-1 and RR-2) is highlighted

More than 50 cuticle and tubulin-related DEGs are included in the “structural constituent of cuticle” and “microtubule-based process” GO terms. For these genes, the following annotations were found: cuticle protein, larval cuticle protein, calcified cuticle protein, pro-resilin, arthrodial cuticle protein, and tubulin protein. In phylogenies, the Tubulin genes of *C. sapidus* were simply assigned with their respective counterparts into two alpha and beta clades *TUBA1* and *TUBB1*, consistent with their annotation (Additional file 3G). Phylogenetic analysis of 10 cuticle proteins from *C. sapidus* yielded three major clades: (1) the CP15.0 sequence was clustered into a separate clade; (2) the AMP8.1 with larval cuticle protein (LCP17-like), AMP1A-like, and then CP8.5; (3) (Fig. 6A) while the last clade included CP6, CP7-like, CP8-like, ACP20, and pro-resilin (Fig. 6C). The cuticle gene phylogenies were supported by strong bootstrap values (83–100 to 100% support) (Additional file 3 H). Sequence analysis showed that the proteins belonging to the latter two clades contain the Rebers-Riddiford 1 or 2 (RR) consensus sequence (58). The RR-1 consensus was present in AMP1A-like, AMP8.1, LCP17-like, and CP8.5 proteins (Fig. 6B); the RR-2 consensus was found in CP6-like, CP7-like, CP8-like, ACP20, and pro-resilin (Fig. 6D), and CP15 contained no detectable RR consensus.

Based on differential expression, 10 cuticle and two tubulin genes were selected by combining the TPM and log2-fold change values (Table 3). Among them, 10 cuticle and tubulin alpha-1 chain (*TUBA1*) genes displayed similar expression patterns, starting from low values in the OE library, then much higher values in the OL library, and then lower values in the AO library. The tubulin beta-1 chain gene (*TUBB1*) showed a different expression pattern, with the highest in the OE library and a gradual decrease from OE to AO.

### Cuticle gene expression based on RNA-seq mapping to the genome

**Fig. 7:**
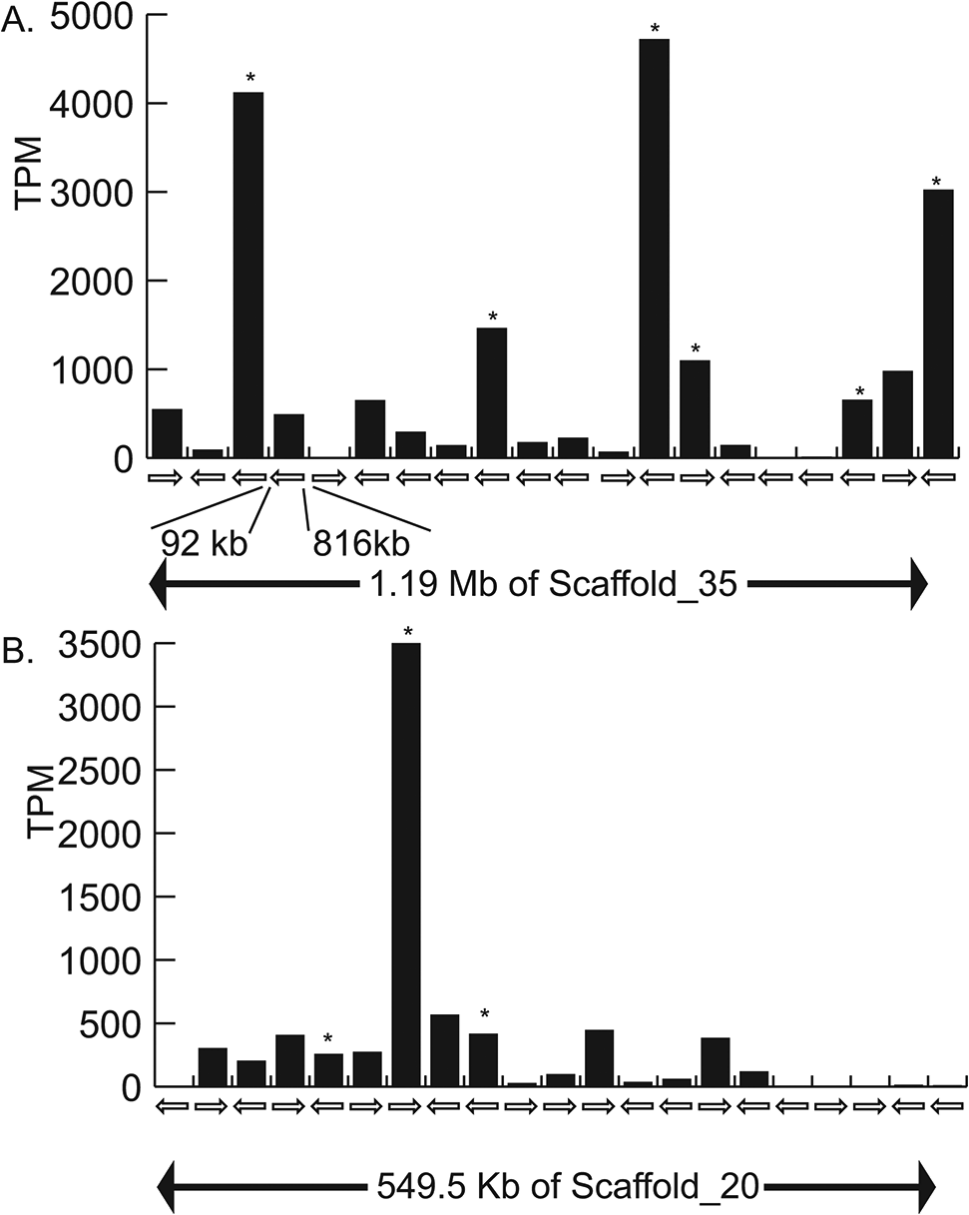
Schematic gene arrangement and expression pattern of the 20 CP-7 like **(A)** and 21 AMP1-like **(B)** cuticle genes and in *C. sapidus.***(A)** A schematic of a 1.19 Mb region of Scaffold 35 is shown with cuticle gene orientation including the location of two large gaps between sets of genes. Expression of these genes during the ovigerous late premolt stage is shown using relative abundance TPM values based on mapping to the genome. An asterisk is used to identify 6 genomic sequences found in the Trinity de novo assembly. **(B)** The 21 AMP1-like cuticular genes in *C. sapidus*. A schematic of a 549.5 Kb region of Scaffold 20 is shown including cuticle gene orientation. Expression of these genes during the Ovigerous Late stage is estimate using relative abundance TPM values based on mapping to the genome. An asterisk is used to identify the three genomic sequences found in the Trinity *de novo* assembly

The CP7-like cuticle proteins were far more highly expressed than any other nine types of cuticle genes in the ovigerous late library. The *CP7-like* relative abundance TPM values were 14,684.52 in the estimate of expression based on Trinity *de novo* assembly and 15,067.56 when using RNA-seq mapping to the genome (Fig. 7A). This single Trinity gene contained 18 isoforms (sequence variants) which in turn were found in the genome with high identity nucleotide (98–100%) matches to six predicted genes and the TPM of these six genes was used to create the overall TPM value. Further examination of this genome region showed this 1.18 Mb section of HiC_scaffold 35 (CM035209.1: 1.26 to 2.44 Mb) contained 20 different CP7-like cuticular genes arranged into two major clusters with gaps of 92 kb and 816 kb between them as shown schematically in Fig. 7A. Of these 21 genes, three were much more highly expressed than the others, with TPM values over 3,000 and orientation between genes switched between the plus strand and minus strand. When examining the *P. trituberculatus* genome assembly, a similar region was found in chromosome 10 (NC_059264.1:11,582 to 11,645 b) containing at least 23 distinct non-overlapping cuticle protein annotations with all oriented in the same direction.

The AMP-1-like set of cuticle proteins were the second most highly expressed type of cuticle protein transcripts in the ovigerous late premolt dataset. Similar to the CP7-like category described above, for AMP-1 there were three Trinity isoforms in the *de novo* assembly which had high (100 to 88% identity) to a set of 21 predicted transcripts from a 550 kb region of HiC_scaffold_20 (CM035194.1: 23.83 to 24.38 Mb) (Fig. 7B). In contrast to the CP7-like cuticle genes shown in Fig. 7A, the AMP-1-like cuticle genes, there was only one dominant transcript expressed in the ovigerous late (OL) stage Figure Y. As with the CP7 example, the *P. trituberculatus* chromosome 44 (NC_059264.1) contained a region of cuticle proteins similar to AMP-1 (~ 90% amino acid identity) from 11.54 to 11.73 Mb.

### RT-qPCR confirmation

**Fig. 8:**
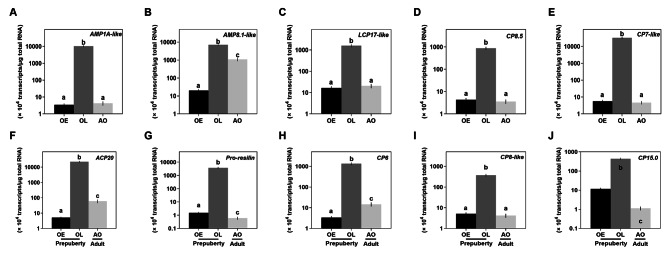
Expression profiles of cuticle genes in the ovigerous setae at different stages by RT-qPCR analysis **(A-J)**. The data are represented as mean ± SE (*n* = 4–5) transcripts/µg total RNA. Statistical significance of the RT-qPCR data were determined using one-way ANOVA followed by Dunn’s posthoc test. Different lowercase letters show significance at *p* ˂ 0.05. Abbreviation: ovigerous setae in the prepuberty females at early (OE) and late premolt (OL) and adult (AO) stages

The RT-qPCR results showed that all the cuticle genes tested were highly expressed in the prepuberty females at late premolt, and a “down-up-down” expression pattern was observed during the ovigerous development from OE to OL and then AO stages (Fig. 8A-J). The *AMP1A-like* and *CP7* cuticle genes were most significantly (*p* < 0.05) changed cuticle transcripts from prepuberty early to premolt stages. Of the five RR-1 consensus sequence gene types defined in Table 3, the *AMP-1 A* gene expression (Fig. 8A) changed the highest from 3.4 ± 0.7 × 10^4^ transcripts/µg total RNA during the OE stage (*n* = 5) to 1.0 ± 0.1 × 10^8^ transcripts/µg total RNA at the OL stage (*n* = 5) and then back down to 4.3 ± 1.2 × 10^4^ transcripts/µg total RNA (*n* = 5) for the Adult stage. The *AMP8.1-like*, *LCP17-like* and *CP8.5-like* had slightly less dramatic changes, while AMP8.1 remained relatively high in the AO stage when the other two were kept low (Fig. 8B-D).

Of the four RR-2 consensus sequences containing cuticle protein genes, the *CP7-like, ACP20*, and *Pro-resilin* transcripts were most significantly changed (Fig. 8E-G). *CP7-like* levels and *Pro-resilin* increased by more than 5,000 fold, the highest levels in the prepuberty females at OL stage to 3.2 ± 0.5 × 10^8^ and 2.2 ± 0.3 × 10^8^ transcripts/µg total RNA (*n* = 5) from 5.6 ± 1.1 × 10^4^ transcripts/µg total RNA (*n* = 5) and 4.1 ± 0.6 × 10^4^ transcripts/µg total RNA (*n* = 5) at OE stage and dropped ~ 6,000 fold to 4.7 ± 1.0 × 10^4^ transcripts/µg total RNA (*n* = 4) and 0.6 ± 0.1 × 10^4^ transcripts/µg total RNA (*n* = 4) in adult (AO) stage (Fig. 8E-G). The *CP6, CP8-like*, and *CP15.0* showed less dramatic changes, with CP6 remaining higher in the AO stage (Fig. 8H) than the other two (Fig. 8I-J).

### Characterization of 5’ flanking sequences of cuticle and tubulin genes

**Fig. 9:**
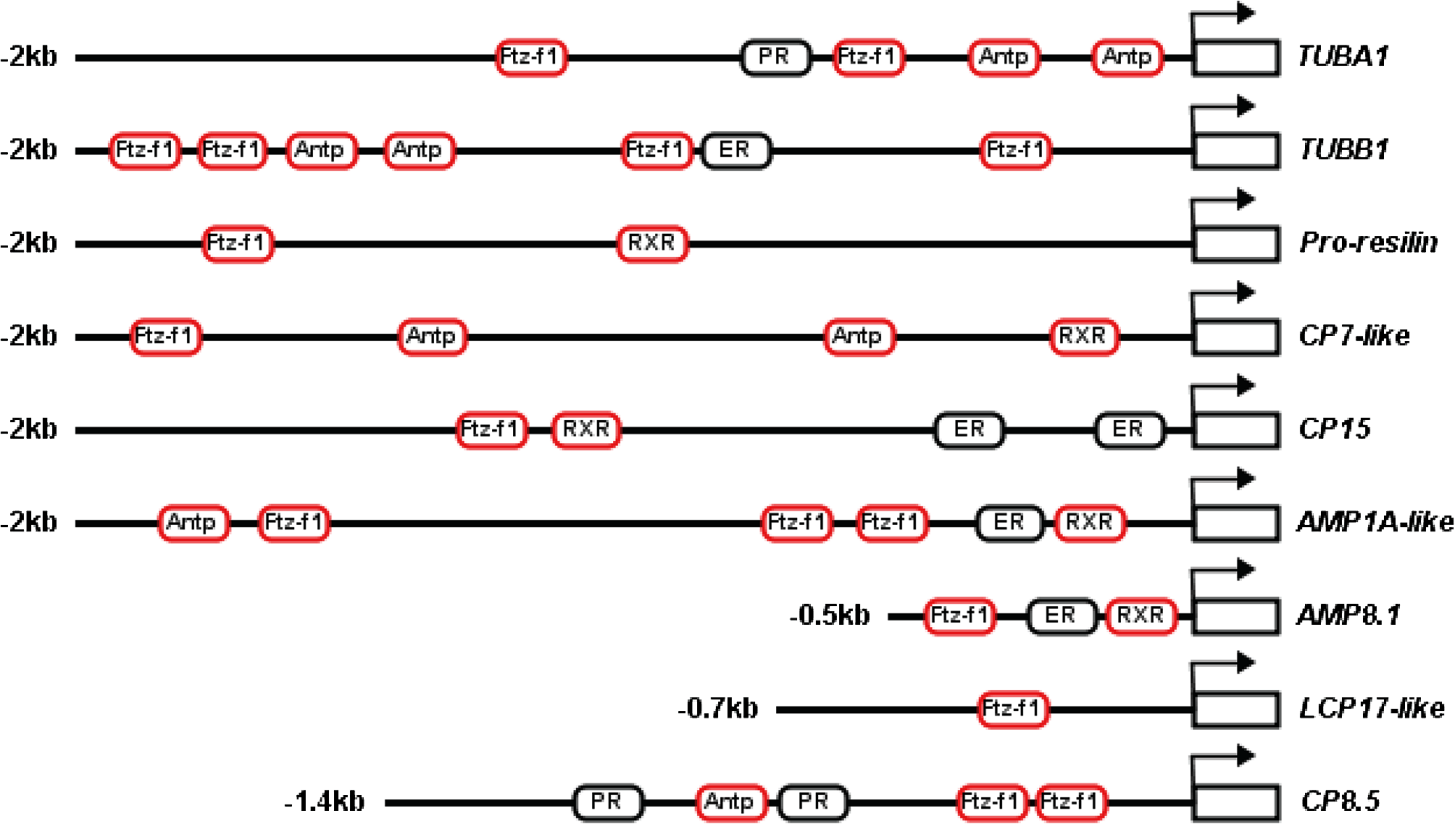
Predicted transcriptional factor-binding sites on the 5’ flanking sequence of cuticle and tubulin genes. The bent arrow indicates the transcription start site (TSS). The putative binding sites for ecdysteroid-responsive factors are marked in red frame. Abbreviation: tubulin alpha-1 chain (*TUBA1*); tubulin beta-1 chain (*TUBB1*); cuticle protein 7-like (*CP7-like*); calcified cuticle protein CP15.0 (*CP15.0*); arthrodial cuticle protein AMP1A-like (*AMP1A-like*); arthrodial cuticle protein AMP8.1 (*AMP8.1*); larval cuticle protein LCP17-like (*LCP17-like*); and calcified cuticle protein CP8.5 (*CP8.5*); Fushi tarazu factor 1 (*Ftz-f1*), Antennapedia (*Antp*), Retinoid X receptor (*RXR*), estrogen receptor (*ER*), and progesterone receptor (*PR*).

An up to 2 kb sequence of the 5’ flanking region of cuticle and tubulin genes was analyzed (Fig. 9). Following putative transcription factor binding sites were identified, including Fushi tarazu factor 1 (Ftz-f1), Antennapedia (Antp), Retinoid X receptor (RXR), estrogen receptor (ER), and progesterone receptor (PR). However, the 5’ flanking region of *CP6*, *CP8-like*, and *ACP20* was not found in the reference genome of *C. sapidus*.

## Discussion

The present study describes the identification of the genes involved in the ovigerous setae development that are essential for carrying the embryos of the animal group Pleocyemata to increase the survival of the offspring. Hence, the genes responsible for these setae development at the prepuberty molt cycle are identified by DEG analysis that may be affected by *C. sapidus* CFSH.

Animals in the phylum Arthropoda have an exoskeleton encasing the entire body. Pleocyemata females develop ovigerous hairs to attach and incubate fertilized eggs while exhibiting extensive brood care behavior. Obviously, oxygen availability differs with the location of the embryo within the egg mass [37–39]. For example, the embryos located deep in the egg mass have less oxygen available than those on the surface. It is known that the flapping of the abdomen of brooding females [39] and the fanning of the ovigerous setae [2, 40, 41] ventilate the embryos, increasing oxygen availability for successful embryogenesis.

Next-generation sequencing was used to describe the *de novo* assembly and annotation of the blue crab, *C. sapidus*, ovigerous setae transcriptomes in prepuberty females at early (OE) and late premolt (OL) and adult (AO) stages regulated by the CFSH. Specifically, cuticle and tubulin transcripts, essential for developing ovigerous hairs are identified with the genes involved in the Wnt signaling and cell cycle.

The quality and completeness of the *de novo* ovigerous setae transcriptome assembly of the pooled RNA samples at the same stage is demonstrated by 95% complete arthropod BUSCOs. Clustering with CD-HIT-EST increases single-copy and reduces duplicate BUSCOs, supporting a clustering strategy. These results and tendencies are comparable to BUSCO outputs generated for other decapod crustaceans [20, 42, 43]. Interestingly, the N50 length of this assembly is similar to those observed for *S. paramamosain* [44] and *Metapenaeus bennettae* [45]. Overall, the number of transcripts in the OL and AO libraries increased about 2-fold compared to OE, indicating high transcriptional diversity.

Each set of comparison generates numerous DEGs. The KEGG enrichment analysis further specifies that the genes involved in the ribosome pathway are the most abundantly upregulated in the AO when comparing the OL vs. AO and OE vs. AO. Ribosomes are the intracellular organelle responsible for protein synthesis [46]. Hence, increased ribosomal protein expression indicates protein synthesis and cellular activity, such as cell differentiation and proliferation [42]. In this study, 40 and 104 DEGs are enriched in the ribosome pathway in the OL vs. AO and OE vs. AO comparisons, respectively, which indicates a dramatic increase in protein synthetic activity in the AO library. Hence, the increases in ribosomal protein synthetic activity is expected as it is essential for the extensive regeneration activity required during each spawning cycle.

Female blue crabs spawn multiple times during a single reproductive season. At each spawning, the female has to regenerate the cuticlular embryonic attachment system, including the egg envelope, the funiculus, and the investment coat has to be newly developed [2]. Hence, the increases in ribosomal protein synthetic activity is expected as it is essential for ovigerous hair formation at OL and regeneration at AO.

Highly enriched DEGs are found in the mTOR signaling, Wnt signaling, and cell cycle pathways. The genes involved in translational machinery such as*S6 kinase* (*S6K*), *unc-51 like autophagy activating kinase 1* (*ULK1/ATG1*), *eukaryotic initiation factor 4E-binding protein 1* (*4E-BP1*), and *eukaryotic translation initiation factor 4E* (*eIF4E*) are highly expressed at the OL stage (Additional file 4), supporting the protein synthesis required for hair formation at this stage as described previously [13]. The increased programmed cell death-related genes in the ovigerous setae at the OL stage may shape the formation of ovigerous hairs. The ovigerous setae transcriptome harbors most key members of the Wnt signaling and cell cycle pathways with OL showing the highest transcript levels of *Wnt2, Wnt5, Wnt6*, *Wnt7, Wnt11*, *Wnt16*, *Frizzled*, *LRP5/6*, *CK1*, *β-catenin*, *AXIN1*, *GSK3β*, *cyclin A*, *cyclin H*, and *CDK7*. Most interestingly, the AO transcriptome, produced from animals at the intermolt stage, shows the highest *cyclin B* and *CDK1* transcripts. This elevated transcript pattern predicates the hair formation during the adult stage as the females spawn multiple times during adulthood without molting [47].

Cytoskeletal gene expression intertwines with cell division and growth. In crustaceans, the hypodermis shows a molt stage-dependent expression pattern with the highest levels of tubulin transcripts at the premolt of *Procambarus clarkii* [48]. Like an insect epidermis, the crustacean hypodermis secretes the epicuticle at the premolt stage as a component of the new exoskeleton. This study does not include the histology of ovigerous setae. However, it is suggested that ovigerous setae may undergo a physiological process similar to molting in the hypodermis. Indeed, cytoskeletal genes, *TUBA1* and *TUBB1* transcript levels peak at the OL stage, the same pattern shown in the crayfish hypodermis at late premolt [48].

The exoskeletal cuticle is rigid and stiff during most of the molt cycle, primarily protecting the animals from an invasion of pathogens. Its softness and flexibility last briefly during and immediately after molting, allowing for molt-related somatic growth of crustaceans [49, 50]. On the other hand, the arthrodial membrane between the main body and legs is membranous and soft, rendering flexible leg movement. Therefore, it is plausible to suggest that differential cuticle protein components are critical for providing a rigid and strong exoskeleton while also providing the proteins for plastic or flexible exoskeleton parts in the tissues like arthrodial membranes and ovigerous hairs.

In contrast to the insect exoskeleton, crustaceans have a calcified exoskeleton containing two different types of cuticle proteins: the cuticle 1 domain potentially associated with cuticle calcification [51] and the chitin_bind_4 domain containing the RR-1 for soft cuticle and − 2 for rigid cuticle [52–54]. Interestingly, putative amino acid sequences of cuticle transcripts exhibiting the cuticle RR-1 domain show no similarity to insect cuticle [55]. Except for CP15.0, all cuticle transcripts contain the chitin_bind_4 domain in the ovigerous setae producing the ovigerous hairs. Clearly, there is a molt-stage dependent expression pattern: LCP17-like, AMP1A-like, and AMP8.1 containing RR-1 domain are expressed at the early premolt stage, possibly providing soft endocuticle and the rest of cuticle transcripts containing the RR-2 domain are expressed at the late premolt stage, possibly sclerotized exocuticle as found in a mosquito, *Anopheles gambie* [55]. Considering the total number of these cuticle transcripts required to form ovigerous hairs, CP7-like, CP8-like, ACP20, and Pro-resilin containing RR-2 domain contribute ~ 75% of transcripts, while those with the RR-1 domain contribute ~ 25%. The ratio between these two RR domains may determine cuticle stiffness. Moreover, further study of the location of these cuticle proteins during ovigerous hair development and the role of CFSH in each cuticle expression.

Given the stimulatory effect of ecdysteroids on molting, a possible explanation for the molt-stage dependent expression pattern of cuticle and tubulin genes in the ovigerous setae is that their transcription may be regulated by ecdysteroid signals. Indeed, the sequence of the upstream promoter region of these genes contains the putative DNA binding sites for several ecdysteroid-responsive factors [56], including *Ftz-f1*, *Antp*, and *RXR*. Two putative transcriptional factor-binding sites, ER and PR, exist in the 5’ flanking sequence of *CP15.0*, *AMP1A-like*, AMP8.1, and *TUBB1*, and *TUBA1* and *CP8.5*, respectively. In vertebrates, ER and PR transfer the effects of estrogen and progesterone on female reproductive physiology by interacting with a DNA-binding domain in receptors with corresponding sex steroid response elements of target genes, respectively [57, 58]. Thus, ER and PR found in the 5’ flanking sequence may imply the regulatory role of vertebrate-type sex hormones in the expression of cuticle and tubulin transcripts.

With the blue crab reference genome available [24], the expansion or contraction of cuticle protein gene families are revealed by their copy numbers and arrangement across different chromosome sized scaffolds. In general, the copy number of these genes is rarely conserved across a broad spectrum of decapods from crabs to shrimps, varying from 1 to 52 among different species (Additional file 1). The dominant OL cuticle transcripts for the RR-1 and RR-2 types were both in gene sets with 21 copies on specific scaffolds, had high nucleotide identity between copies, shared a similar chromosomal arrangement with *P. trituberculatus*, and individual copies were selectively expressed in this tissue at the late premolt stage. For example, the most highly expressed cuticle gene set was the *CP7-like* type and only three specific *loci* were very highly expressed during the ovigerous late stage. More specifically in AMP-1 which was the second-most abundantly expressed cuticle protein set, only a single gene was highly transcribed from within the large array on chromosome 20.

These data suggest strong transcriptional selection of specific gene copies in the OL tissues, from amongst the large available set of similar cuticle proteins. Overall, the highly contributing proteins to the ovigerous hair likely are the CP-7-like and AMP1 cuticle proteins and within those categories only specific subtypes are selected for enhanced transcription at this life stage. Their tandem arrangement in scaffolds suggests that the additional gene copy may have arisen through a small-scale duplication event, while those overlapping functional genes situated on different scaffolds may arise from a species-specific whole-genome duplication event [59]. Interestingly, the discrepancy in the TPM value of two tandemly distributed *pro-resilin* (Chr_9_Csap_19436 and 19,438) is noted in that Chr_9_Csap_19436 is ~ 108 fold more expressed than 19,438 (Additional excel.file 1). Such a difference is possibly caused by the ER and PR binding sites and superiority in the number of binding sites for ecdysteroid-responsive factors (Additional file 5), which may provide additional evidence for the importance of ecdysteroid and sex steroids in the regulation of cuticle gene transcription.

**Fig. 10:**
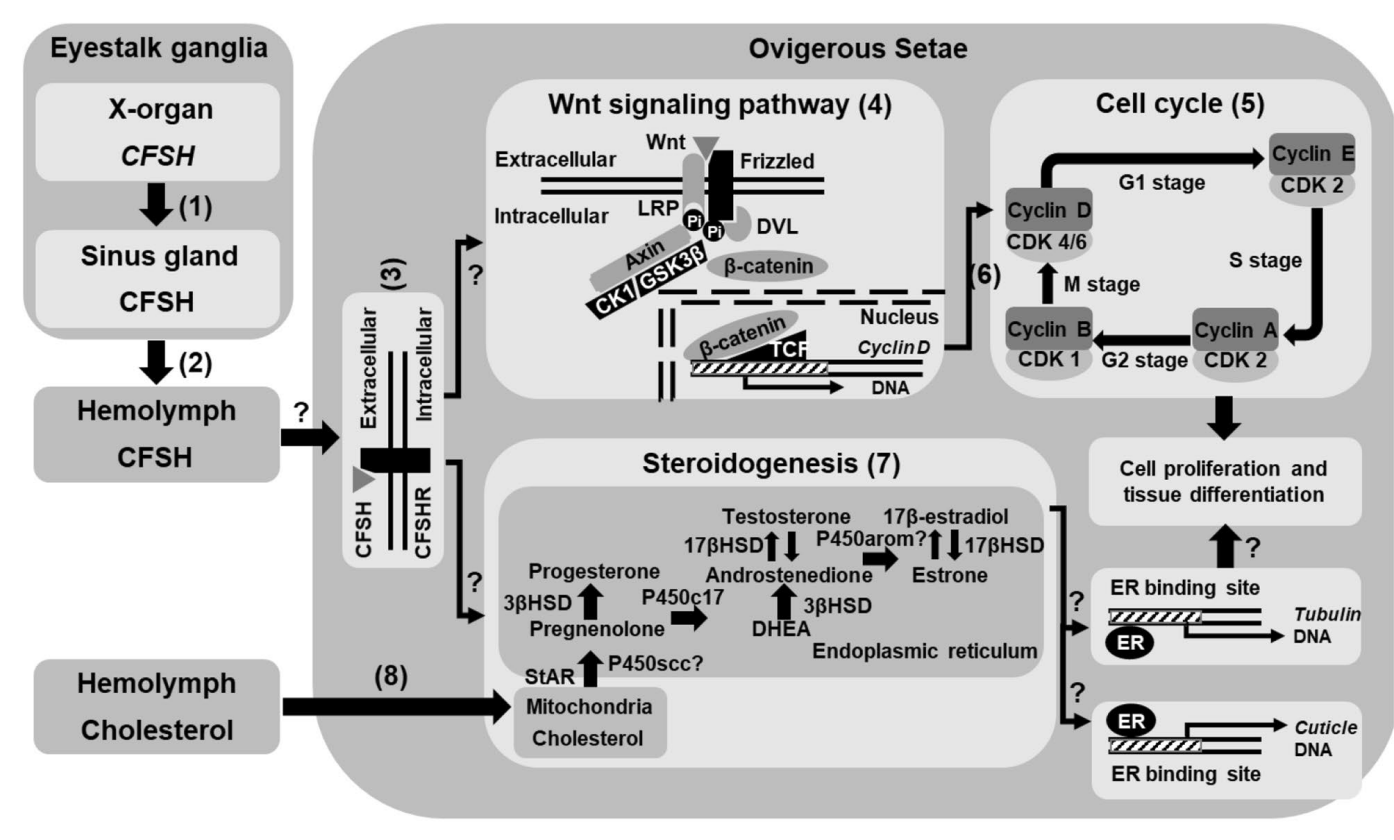
Proposed, putative mode of CFSH action in crustaceans based on the previous and present findings. First, CFSH is produced in the X-organ, temporally stored in the sinus glands, and released into the hemolymph. Then, binding of CFSH to CFSHR activates the intracellular signal transduction factors, which further triggers the rapid activation of the Wnt signaling and steroidogenesis. In the Wnt signaling cascade, the activated Wnt ligands bind to Frizzled and LRP6 receptors, leading to the phosphorylation of LRP6 and the disassembly of the destruction complex (DVL/AXIN/GSK3β/CK1/β-catenin), allowing the release of the stabilized β-catenin to cytoplasm. The free β-catenin is subsequently translocated into cell nucleus, stimulating *cyclin D* expression in concert with TCF. The up-regulated *cyclin D* expression promotes cell cycle progression, cell proliferation and tissue differentiation. On the other hand, with the increase in hemolymph cholesterol level, CFSH may increase E2 synthesis and release in the ovigerous setae via up-regulating *StAR3* expression. The elevated E2 levels may trigger tissue differentiation via activating the transcription of cuticle and tubulin genes. The identification of CFSHR and CFSH signal transduction factors, the regulatory interaction among CFSH, the Wnt signaling and cell cycle pathways, and cuticle and tubulin genes, and ‘?’ requiring a further study. Abbreviations: TCF (T-cell factor), P450scc (P450 side-chain cleavage); *StAR3* (StAR-related lipid transfer protein 3); 3βHSD (3β-hydroxysteroid dehydrogenase); P450c17 (17α-hydroxylase/17, 20-lyase); 17βHSD (17β-hydroxysteroid dehydrogenase); P450arom (P450 aromatase); and DHEA (dehydroepiandrosterone)

Cuticle and tubulin-related transcripts and genes involved in the Wnt signaling and cell cycle were demonstrated here to be essential for developing ovigerous hairs. Combining these findings with previous ones in the literature enabled the construction of **a putative mode** of CFSH triggering pathway of ovigerous hair formation in decapod crustaceans, using *C. sapidus* as a model (Fig. 10). First, CFSH is produced in the eyestalk ganglia and released into the hemolymph [7, 13]. Next, binding of CFSH to its putative receptor (CFSHR) stimulates levels of CFSH second messenger, which further triggers rapid activation of the Wnt signaling cascade and sex steroid production. The activated Wnt signaling pathway up-regulates downstream *cyclin D* transcription, which drives cell cycle progression, cell proliferation, and ovigerous hair formation. On the other hand, the increased hemolymph cholesterol level [60] and StAR-related lipid transfer protein 3 (*StAR3*) expression that is positively regulated by CFSH [14] result in a significant rise in E2 levels in the ovigerous setae, which further induce ovigerous hair formation via activating the transcription of cuticle and tubulin genes.

To date, the CFSH receptor and its potential second messenger have not been identified in crustacean species. The direct effects of CFSH on Wnt signaling and cell cycle, and cuticle and tubulin gene expression remain to be studied for the interpretation of mode of CFSH action in crustaceans. Moreover, based on the enhanced cell cycle progression and proliferation in the prepuberty females at late premolt, studies for histological analysis are also in progress with adult-female-specific feature development.

## Conclusions

The present study represents a first step in understanding the mechanism underlying ovigerous hair formation in *C. sapidus* at the molecular level. The Wnt signaling and cell cycle pathways, as well as cuticle and tubulin genes may play essential roles in ovigerous hair development. In addition, the putative mode of CFSH action in crustaceans is proposed based on the present and previous findings. However, the obtained results are preliminary, and the precise functional roles of the positively enriched pathways and DEGs in developing adult female-specific tissues require further confirmation and investigation. Overall, the present study depicts the first transcriptome analysis of crustacean ovigerous setae and the results will be helpful for future studies on female reproductive biology in *C. sapidus*.

### Electronic supplementary material

Below is the link to the electronic supplementary material.

Supplementary Material 1

Supplementary Material 2

Supplementary Material 3

Supplementary Material 4

## Data Availability

Raw read sequence files are archived in the NCBI SRA archive under BioProject PRJNA1005288 (https://dataview.ncbi.nlm.nih.gov/object/PRJNA1005288).
