# Supplementary material for: Identifying the genes involved in the egg-carrying ovigerous hair development of the female blue crab Callinectes sapidus: transcriptomic and genomic expression analyses
Source: BMC Genomics. 2023 Dec 11;24:764. doi: 10.1186/s12864-023-09862-9 (PMC10712104; doi:10.1186/s12864-023-09862-9)

Wang et al., Additional file 1

| **A** | **B** |
| --- | --- |
| **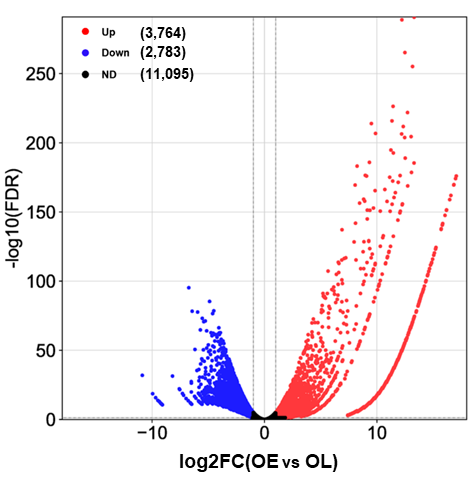** | **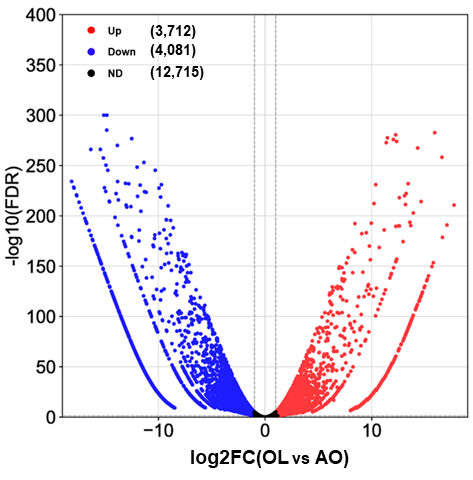** |
| **C** | **D** |
| 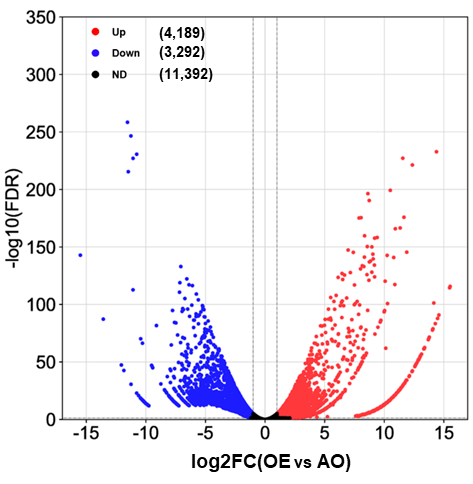 | 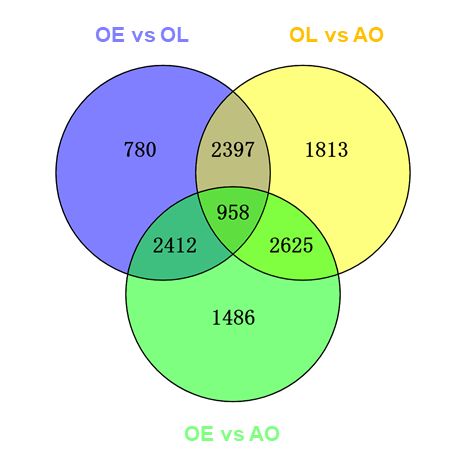 |

Wang et al., Additional file 2

| **A** | **C** |
| --- | --- |
|  | 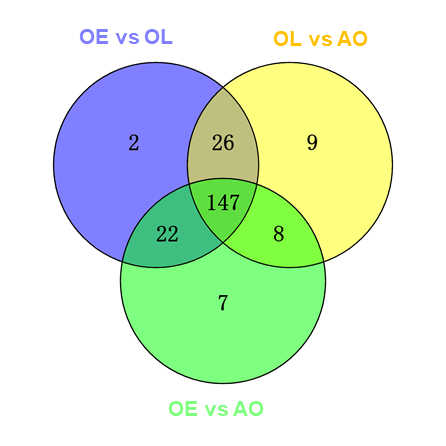 |
| **B** | **D** |
|  | 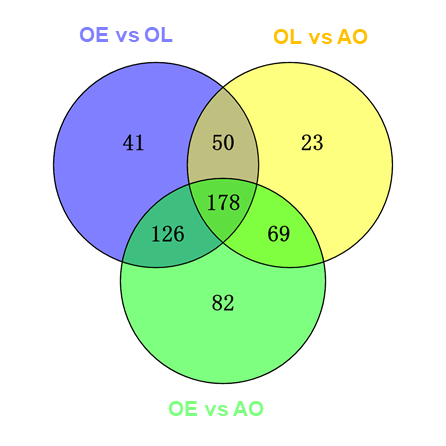 |

Wang et al., Additional file 3

| **A** | **B** |
| --- | --- |
| 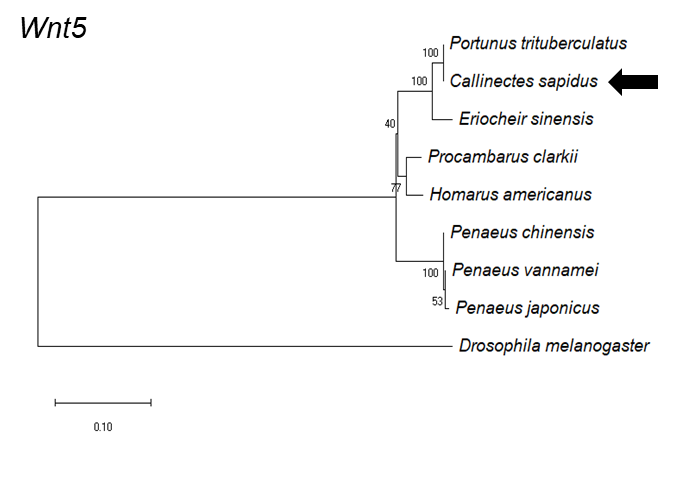 | 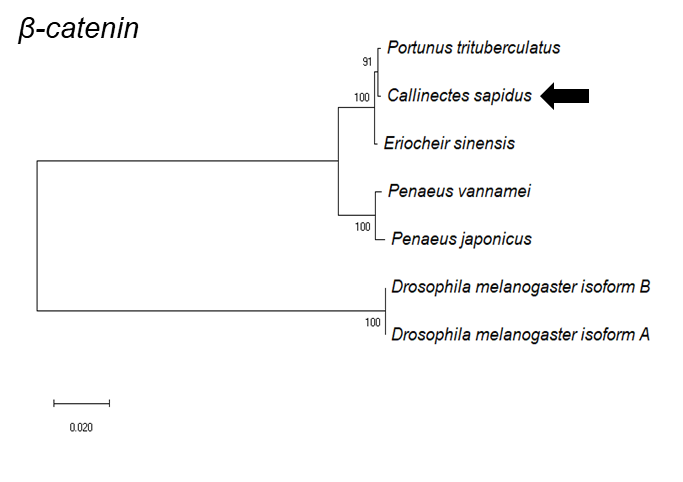 |
| **C** | **D** |
| 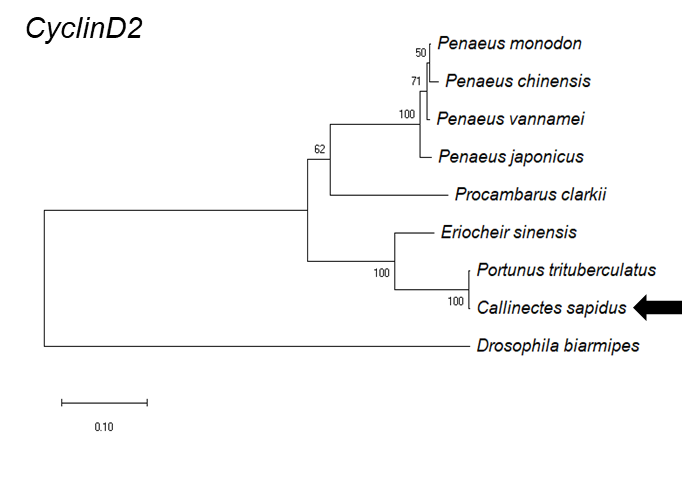 | 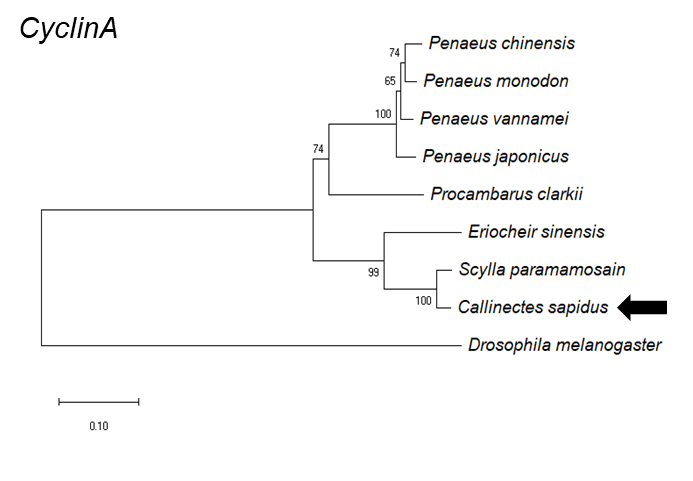 |
| **E** | **F** |
| 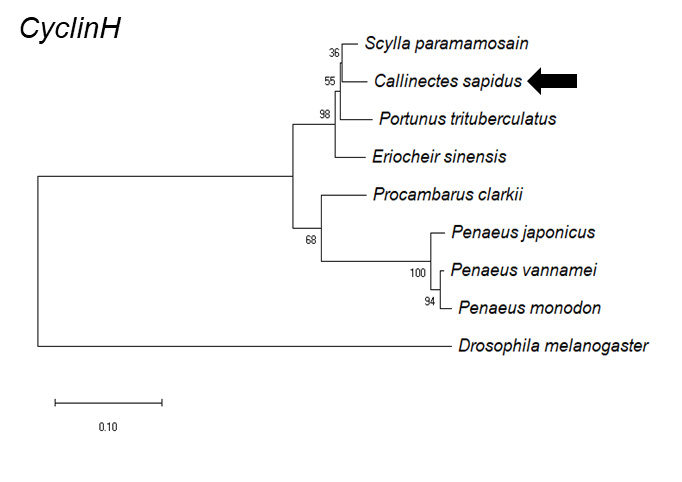 | 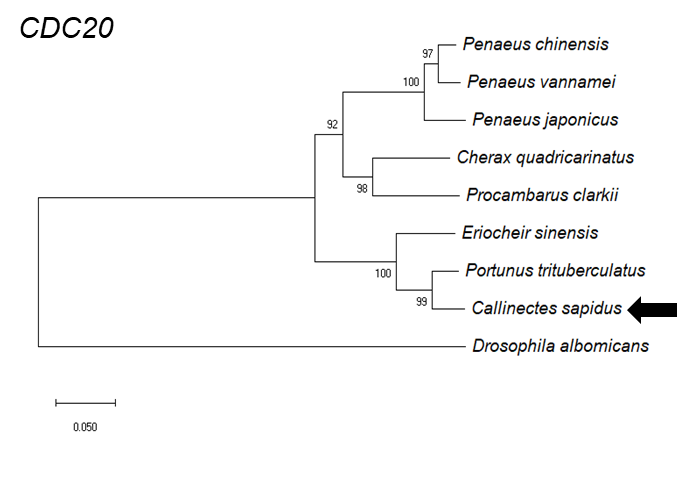 |
| **G** | **H** |
| 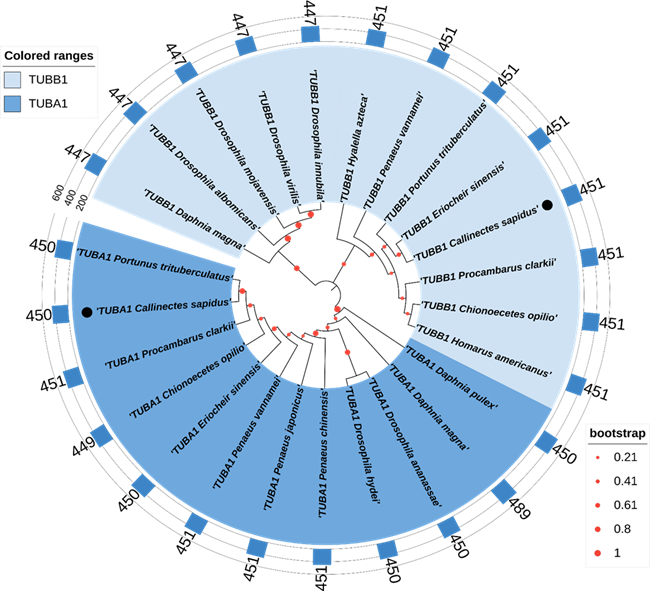 | 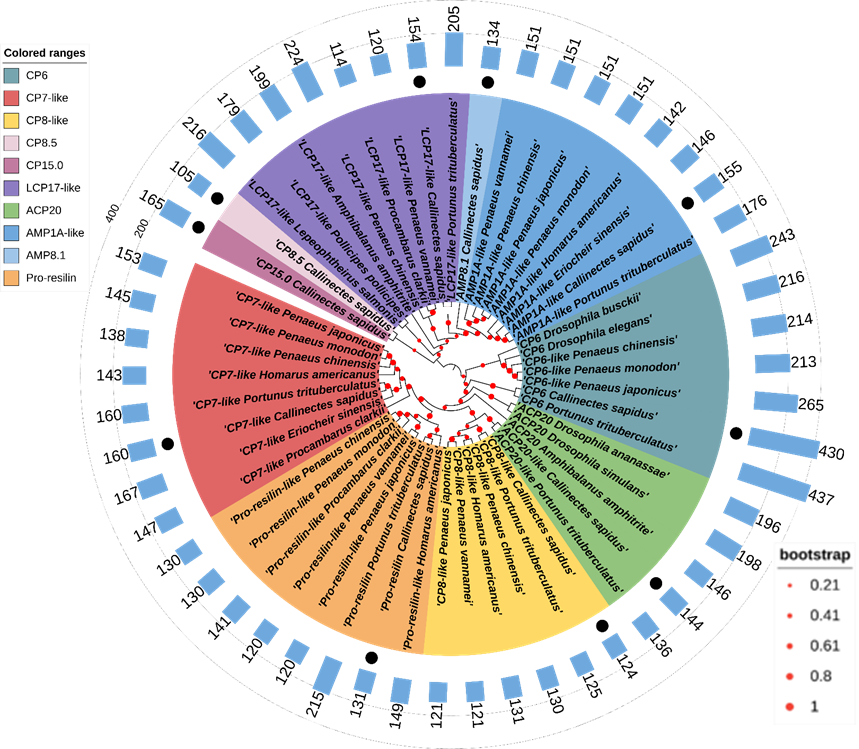 |

Wang et al., Additional file 4

| **A** |
| --- |
| 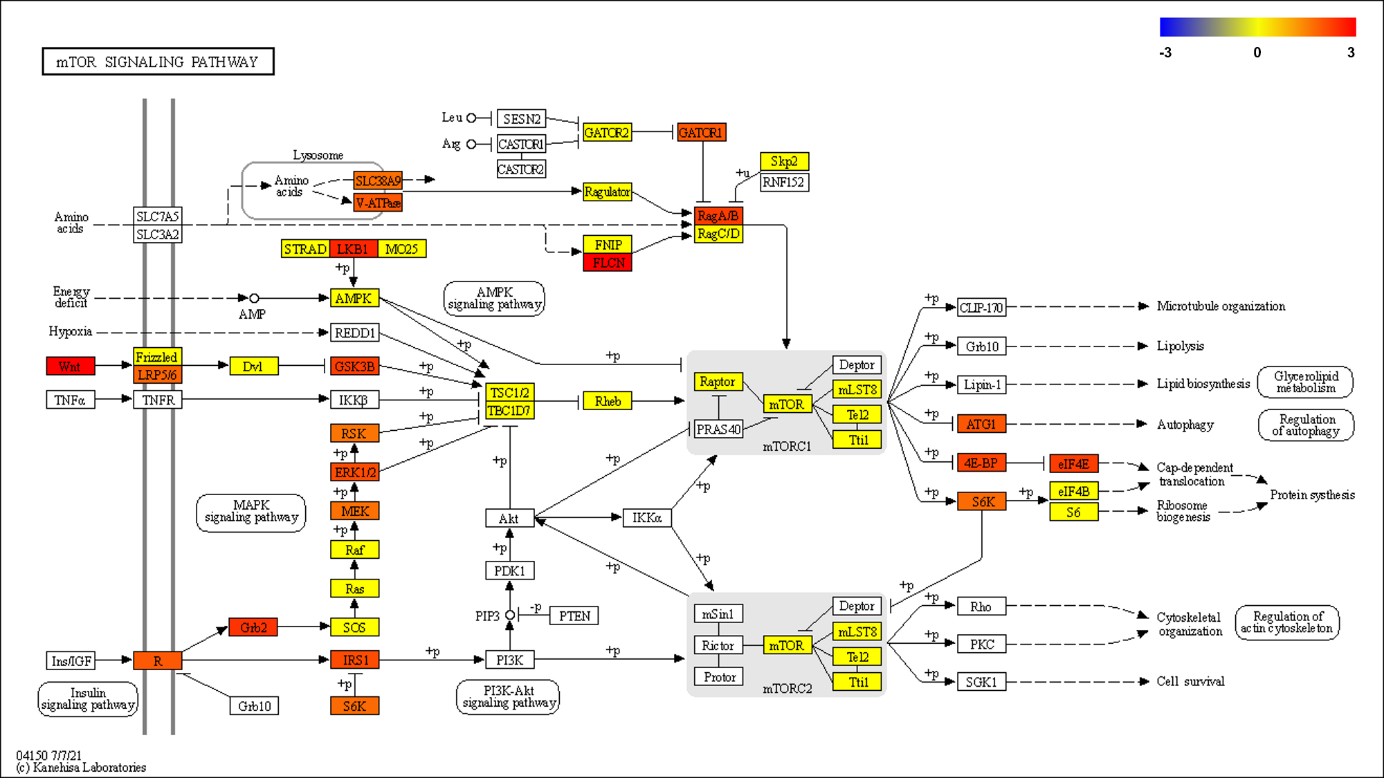 |
| **B** |
| 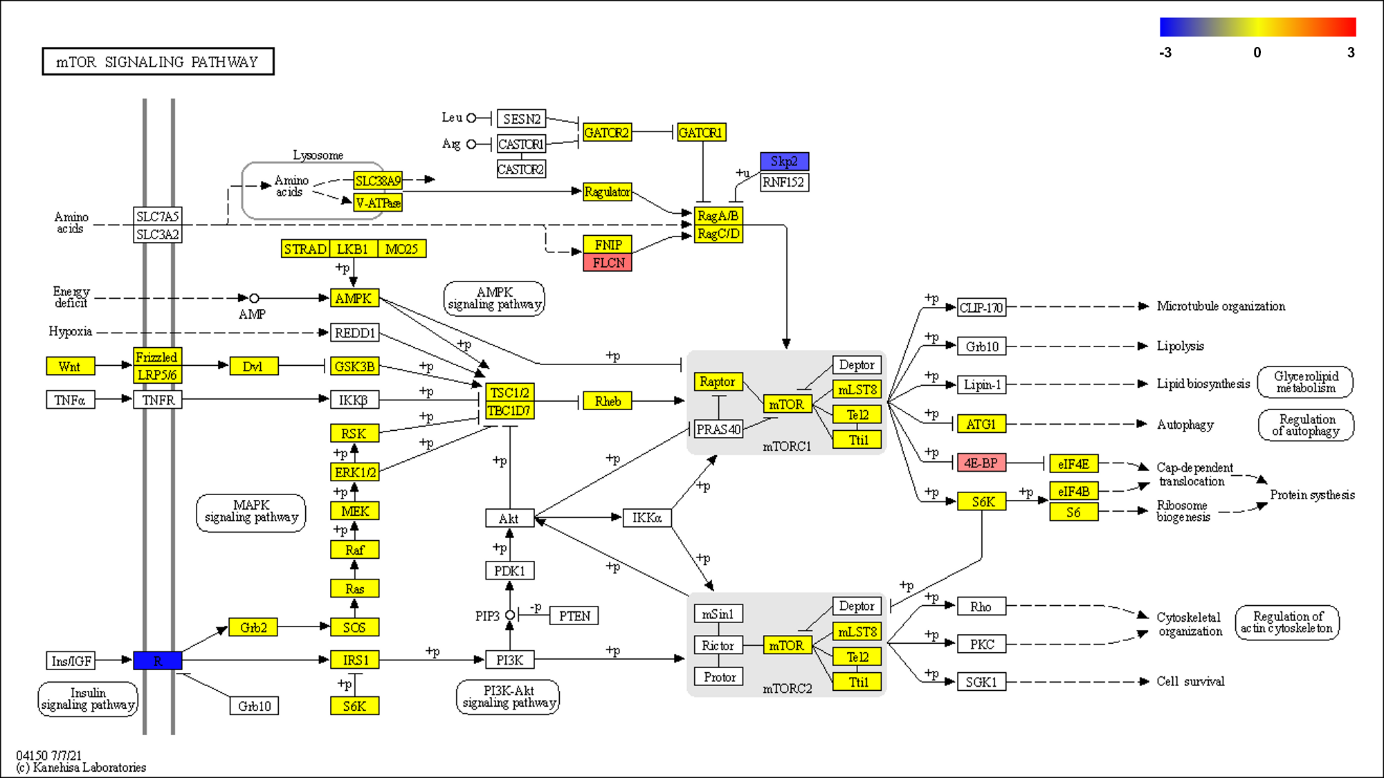 |
| **C** |
| 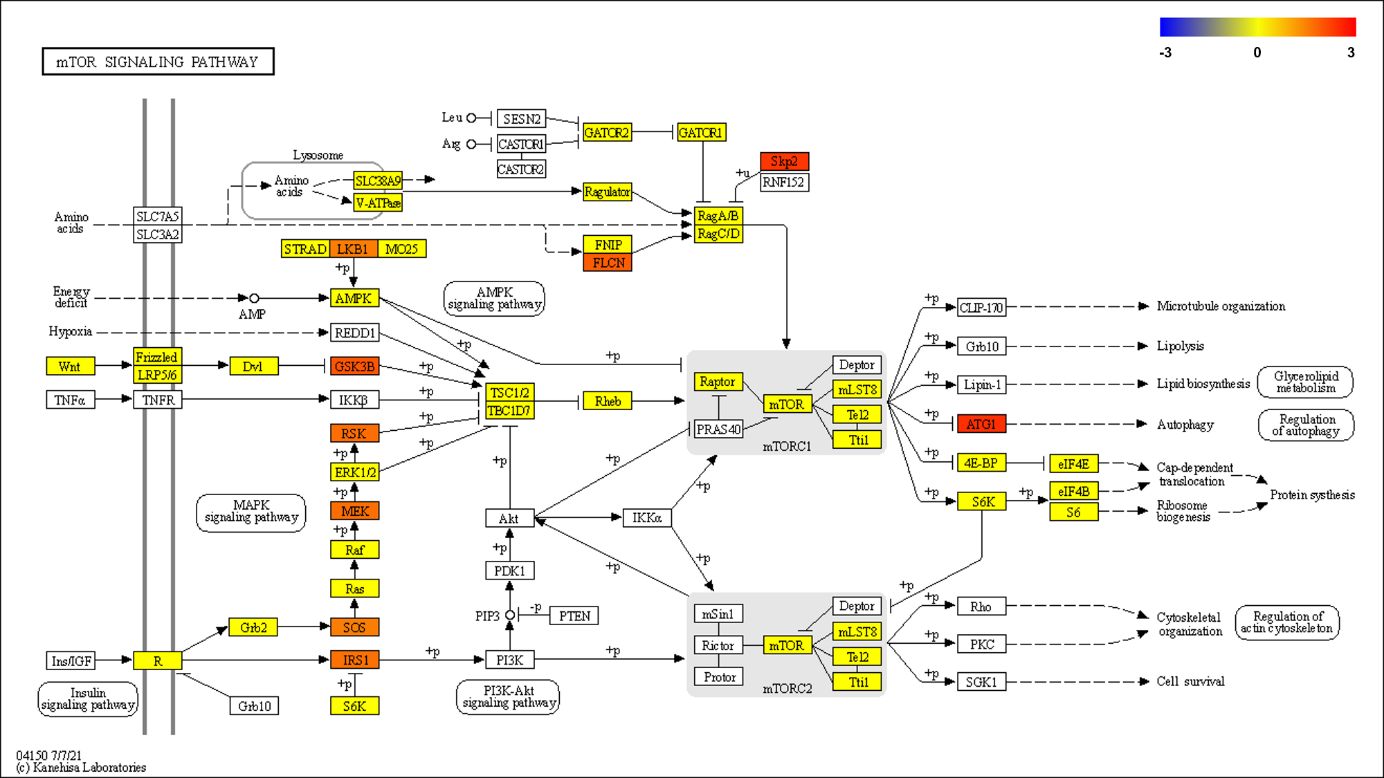 |

Wang et al., Additional file 5


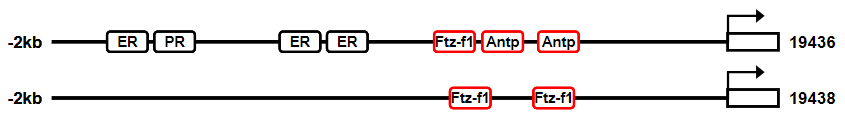

Supplement: Supplementary file 1 — Supplementary Material 1 [file 12864_2023_9862_MOESM1_ESM.docx]
