# Supplementary material for: Identifying the genes involved in the egg-carrying ovigerous hair development of the female blue crab Callinectes sapidus: transcriptomic and genomic expression analyses
Source: BMC Genomics. 2023 Dec 11;24:764. doi: 10.1186/s12864-023-09862-9 (PMC10712104; doi:10.1186/s12864-023-09862-9)
Supplement: Supplementary file 3 — Supplementary Material 3 [file 12864_2023_9862_MOESM3_ESM.docx]

Additional table 1. The information of the sequences used in phylogenetic analysis.

| **Gene name** | ***Species*** | **Accession number** | **Full name** |
| --- | --- | --- | --- |
| *Beta-catenin* | *Drosophila melanogaster* | NP_476666.1 | armadillo, isoform A |
|  | *Drosophila melanogaster* | NP_599100.1 | armadillo, isoform B |
|  | *Eriocheir sinensis* | QKI80084.1 | Beta-catenin |
|  | *Penaeus japonicus* | ANH11481.1 | Beta-catenin |
|  | *Penaeus vannamei* | ALK24421.1 | Beta-catenin |
|  | *Portunus trituberculatus* | WBU15554.1 | Beta-catenin |
|  | *Callinectes sapidus* | TRINITY_DN2531_c0_g1 | Beta-catenin |
| *Wnt* | *Drosophila melanogaster* | AAF52501.1 | wingless |
|  | *Penaeus vannamei* | XP_027212902.1 | protein Wnt-5b-like |
|  | *Penaeus chinensis* | XP_047495064.1 | protein Wnt-5b-like |
|  | *Penaeus japonicus* | XP_042866443.1 | protein Wnt-5b-like |
|  | *Homarus americanus* | XP_042208614.1 | protein Wnt-5b-like |
|  | *Procambarus clarkii* | XP_045616766.1 | protein Wnt-5b-like |
|  | *Eriocheir sinensis* | XP_050726753.1 | protein Wnt-5b-like |
|  | *Portunus trituberculatus* | XP_045131549.1 | protein Wnt-5b-like |
|  | *Callinectes sapidus* | TRINITY_DN12886_c0_g1 | protein Wnt-5b-like |
| *cyclin-D* | *Drosophila biarmipes* | XP_043949395.1 | G1/S-specific cyclin-D2 |
|  | *Procambarus clarkii* | XP_045621437.1 | G1/S-specific cyclin-D2-like |
|  | *Penaeus japonicus* | XP_042883999.1 | G1/S-specific cyclin-D2-like |
|  | *Penaeus chinensis* | XP_047501953.1 | G1/S-specific cyclin-D2-like |
|  | *Penaeus vannamei* | XP_027230171.1 | G1/S-specific cyclin-D2-like |
|  | *Penaeus monodon* | XP_037801165.1 | G1/S-specific cyclin-D2-like |
|  | *Eriocheir sinensis* | XP_050737200.1 | G1/S-specific cyclin-D2-like |
|  | *Portunus trituberculatus* | XP_045103140.1 | G1/S-specific cyclin-D2-like |
|  | *Callinectes sapidus* | TRINITY_DN15149_c0_g1 | G1/S-specific cyclin-D2-like |
| *cyclin-A* | *Drosophila melanogaster* | BAA01629.1 | Cyclin A |
|  | *Penaeus monodon* | XP_037802348.1 | G2/mitotic-specific cyclin-A-like |
|  | *Penaeus vannamei* | XP_027230063.1 | G2/mitotic-specific cyclin-A-like |
|  | *Penaeus chinensis* | XP_047479732.1 | G2/mitotic-specific cyclin-A-like |
|  | *Penaeus japonicus* | XP_042882527.1 | G2/mitotic-specific cyclin-A-like |
|  | *Procambarus clarkii* | XP_045592535.1 | G2/mitotic-specific cyclin-A-like |
|  | *Eriocheir sinensis* | XP_050729457.1 | Cyclin-A2-like |
|  | *Scylla paramamosain* | ADK13092.1 | Cyclin A |
|  | *Callinectes sapidus* | TRINITY_DN9609_c0_g1 | Cyclin A |
| *cyclin-H* | *Drosophila melanogaster* | NP_524207.1 | Cyclin H |
|  | *Penaeus monodon* | AGP03382.1 | Cyclin H |
|  | *Penaeus vannamei* | XP_027215413.1 | Cyclin-H-like |
|  | *Penaeus japonicus* | XP_042887304.1 | Cyclin-H-like |
|  | *Procambarus clarkii* | XP_045602408.1 | Cyclin-H-like |
|  | *Eriocheir sinensis* | XP_050723330.1 | Cyclin-H-like |
|  | *Portunus trituberculatus* | XP_045131721.1 | Cyclin-H-like |
|  | *Scylla paramamosain* | ACL81559.1 | Cyclin H |
|  | *Callinectes sapidus* | TRINITY_DN1669_c1_g1 | Cyclin H |
| *Cdc20* | *Drosophila albomicans* | XP_034100966.1 | cell division cycle protein 20 homolog |
|  | *Penaeus chinensis* | XP_047477592.1 | cell division cycle protein 20 homolog isoform X1 |
|  | *Penaeus japonicus* | XP_042865898.1 | cell division cycle protein 20 homolog isoform X1 |
|  | *Penaeus vannamei* | XP_027225735.1 | cell division cycle protein 20 homolog |
|  | *Procambarus clarkii* | XP_045599016.1 | cell division cycle protein 20 homolog isoform X1 |
|  | *Cherax quadricarinatus* | XP_053644797.1 | cell division cycle protein 20 homolog isoform X1 |
|  | *Eriocheir sinensis* | XP_050690303.1 | cell division cycle protein 20 homolog isoform X1 |
|  | *Portunus trituberculatus* | XP_045123993.1 | cell division cycle protein 20 homolog |
|  | *Callinectes sapidus* | TRINITY_DN24059_c0_g1 | cell division cycle protein 20 |
| *TUBA1* | *Portunus trituberculatus* | XP_045137688.1 | tubulin alpha-1 chain |
|  | *Eriocheir sinensis* | XP_050687740.1 | tubulin alpha-1 chain |
|  | *Procambarus clarkii* | XP_045596715.1 | tubulin alpha-1 chain |
|  | *Chionoecetes opilio* | KAG0727717.1 | tubulin alpha-1 chain |
|  | *Penaeus vannamei* | XP_027223066.1 | tubulin alpha-1 chain |
|  | *Penaeus japonicus* | XP_042887706.1 | tubulin alpha-1 chain |
|  | *Penaeus chinensis* | XP_047492967.1 | tubulin alpha-1 chain |
|  | *Daphnia magna* | XP_032779159.2 | tubulin alpha-1 chain |
|  | *Daphnia pulex* | XP_046438580.1 | tubulin alpha-1 chain |
|  | *Drosophila hydei* | XP_023177012.1 | tubulin alpha-1 chain |
|  | *Drosophila ananassae* | XP_001953205.1 | tubulin alpha-1 chain |
|  | *Callinectes sapidus* | TRINITY_DN19_c0_g1 | tubulin alpha-1 chain |
| *TUBB1* | *Portunus trituberculatus* | XP_045129538.1 | tubulin beta-1 chain |
|  | *Eriocheir sinensis* | XP_050690677.1 | tubulin beta-1 chain |
|  | *Homarus americanus* | XP_042236751.1 | tubulin beta-1 chain |
|  | *Procambarus clarkii* | XP_045597784.1 | tubulin beta-1 chain |
|  | *Chionoecetes opilio* | KAG0715060.1 | tubulin beta-1 chain |
|  | *Penaeus vannamei* | XP_027223912.1 | tubulin beta-1 chain |
|  | *Hyalella azteca* | XP_018017159.1 | tubulin beta-1 chain |
|  | *Daphnia magna* | XP_032777943.1 | tubulin beta-1 chain |
|  | *Drosophila albomicans* | XP_034107023.1 | tubulin beta-1 chain |
|  | *Drosophila mojavensis* | XP_002005898.1 | tubulin beta-1 chain |
|  | *Drosophila innubila* | XP_034476415.1 | tubulin beta-1 chain |
|  | *Drosophila virilis* | XP_002049944.1 | tubulin beta-1 chain |
|  | *Callinectes sapidus* | TRINITY_DN197_c34_g1 | tubulin beta-1 chain |
| CP6 | *Portunus trituberculatus* | MPC43157.1 | Cuticle protein 6 |
|  | *Penaeus japonicus* | XP_042869348.1 | cuticle protein 6-like |
|  | *Penaeus monodon* | XP_037774143.1 | cuticle protein 6-like |
|  | *Penaeus chinensis* | XP_047491675.1 | cuticle protein 6-like |
|  | *Drosophila busckii* | XP_017840211.2 | Cuticle protein 6 |
|  | *Drosophila elegans* | XP_017126656.1 | Cuticle protein 7 |
|  | *Callinectes sapidus* | TRINITY_DN2304_c0_g1 | Cuticle protein 6 |
| CP7-like | *Portunus trituberculatus* | XP_045107367.1 | cuticle protein 7-like |
|  | *Eriocheir sinensis* | XP_050705288.1 | cuticle protein 7-like |
|  | *Homarus americanus* | XP_042239697.1 | cuticle protein 7-like |
|  | *Penaeus japonicus* | XP_042884390.1 | cuticle protein 7-like |
|  | *Penaeus monodon* | XP_037801298.1 | cuticle protein 7-like |
|  | *Procambarus clarkii* | XP_045624460.1 | cuticle protein 7-like |
|  | *Penaeus chinensis* | XP_047472520.1 | cuticle protein 7-like |
|  | *Callinectes sapidus* | TRINITY_DN11_c0_g1 | cuticle protein 7-like |
| CP8-like | *Portunus trituberculatus* | XP_045101387.1 | cuticle protein 8-like |
|  | *Penaeus japonicus* | XP_042861418.1 | cuticle protein 8-like |
|  | *Penaeus vannamei* | XP_027227161.1 | cuticle protein 8-like |
|  | *Penaeus chinensis* | XP_047479534.1 | cuticle protein 8-like |
|  | *Callinectes sapidus* | TRINITY_DN181_c0_g1 | cuticle protein 8-like |
| CP8.5 | *Callinectes sapidus* | AAV28478.1 | calcified cuticle protein CP8.5 |
| CP15.0 | *Callinectes sapidus* | ABB91679.1 | calcified cuticle protein CP15.0 |
| ACP20 | *Portunus trituberculatus* | XP_045101342.1 | adult-specific cuticular protein ACP-20-like |
|  | *Amphibalanus amphitrite* | KAF0310630.1 | adult-specific cuticular protein ACP-20 |
|  | *Drosophila simulans* | XP_002085495.1 | adult-specific cuticular protein ACP-20 |
|  | *Drosophila ananassae* | XP_001958116.1 | adult-specific cuticular protein ACP-20 |
|  | *Callinectes sapidus* | TRINITY_DN1246_c1_g1 | adult-specific cuticular protein ACP-20-like |
| LCP17-like | *Pollicipes pollicipes* | XP_037094443.1 | larval cuticle protein LCP-17-like |
|  | *Penaeus vannamei* | XP_027236378.1 | larval cuticle protein LCP-17-like |
|  | *Penaeus chinensis* | XP_047476033.1 | larval cuticle protein LCP-17-like |
|  | *Procambarus clarkii* | XP_045608559.1 | larval cuticle protein LCP-17-like |
|  | *Portunus trituberculatus* | XP_045101992.1 | larval cuticle protein LCP-17-like |
|  | *Amphibalanus amphitrite* | XP_043209214.1 | larval cuticle protein LCP-17-like |
|  | *Lepeophtheirus salmonis* | XP_040581928.1 | larval cuticle protein LCP-17-like |
|  | *Callinectes sapidus* | TRINITY_DN44_c1_g2 | larval cuticle protein LCP-17-like |
| AMP1A-like | *Portunus trituberculatus* | XP_045136044.1 | arthrodial cuticle protein AMP1A-like |
|  | *Eriocheir sinensis* | XP_050700851.1 | arthrodial cuticle protein AMP1A-like |
|  | *Penaeus monodon* | XP_037780487.1 | arthrodial cuticle protein AMP1A-like |
|  | *Penaeus vannamei* | XP_027209005.1 | arthrodial cuticle protein AMP1A-like |
|  | *Penaeus japonicus* | XP_042891716.1 | arthrodial cuticle protein AMP1A-like |
|  | *Penaeus chinensis* | XP_047469442.1 | arthrodial cuticle protein AMP1A-like |
|  | *Homarus americanus* | XP_042218341.1 | arthrodial cuticle protein AMP1A-like |
|  | *Procambarus clarkii* | XP_045593236.1 | arthrodial cuticle protein AMP1A-like |
|  | *Callinectes sapidus* | TRINITY_DN9_c0_g1 | arthrodial cuticle protein AMP1A-like |
| AMP8.1 | *Callinectes sapidus* | AAV28476.1 | arthrodial cuticle protein AMP8.1 |
| Pro-resilin | *Homarus americanus* | XP_042211318.1 | pro-resilin-like |
|  | *Penaeus chinensis* | XP_047478941.1 | pro-resilin-like |
|  | *Procambarus clarkii* | XP_045617302.1 | pro-resilin-like |
|  | *Penaeus monodon* | XP_037801879.1 | pro-resilin-like |
|  | *Portunus trituberculatus* | MPC57010.1 | pro-resilin |
|  | *Penaeus vannamei* | XP_027213807.1 | pro-resilin-like |
|  | *Penaeus japonicus* | XP_042875665.1 | pro-resilin-like |
|  | *Callinectes sapidus* | TRINITY_DN38_c23_g2 | Pro-resilin |

Additional table 2. Primers used for RT-qPCR in this study.

| **Primers** | **Sequence (5’ - 3’)** | **Size (nt)** | **Efficiency (%)** |
| --- | --- | --- | --- |
| Wnt5b-qF | GCGTGTCTGGGTCTTGTTCT | 103 | 98.4 |
| Wnt5b-qR | ACCTCAGTGGCCCCATCATA |  |  |
| β-catenin-qF | GTCACACCACCGTCAAGGAT | 116 | 90.0 |
| β-catenin-qR | GCAGCGTTGTGATGGCATAG |  |  |
| Cyclin D-qF | CTGGCCGTCATTGAACCAGA | 110 | 95.9 |
| Cyclin D-qR | GCAGCGGTACACTCGGATAA |  |  |
| Cyclin A-qF | TCCATGCACGCACTGATGAT | 103 | 92.7 |
| Cyclin A-qR | TAGTTGGCCCGAGGTTTGTG |  |  |
| Cyclin H-qF | AACCTGTGTCTACCTGGCCT | 94 | 101.2 |
| Cyclin H-qR | AATGGCCTTCTTCCTGTCGC |  |  |
| Cdc20-qF | GCGTCCCTGAGAAGATCCTG | 98 | 97.6 |
| Cdc20-qR | CAGAGCAACTCCCAGATGGT |  |  |
| CP6-qF | ACTCCTTCGGCTACAATGCC | 117 | 94.1 |
| CP6-qR | AGTGCTGCGTCTGTAGGTTG |  |  |
| CP7-like-qF | GTGCCCAGGAGAATCGTGAC | 104 | 90.5 |
| CP7-like-qR | TGAGTCACCGTCCACGTAGT |  |  |
| CP8-like-qF | GTACGGCGTGAAGGATGACT | 102 | 98.7 |
| CP8-like-qR | CCGTCTGGAAGGACCACTTG |  |  |
| AMP1A-like-qF | GGCCTTATAGCTATGGCTCAG | 104 | 93.6 |
| AMP1A-like-qR | GGTCCTCATTTCCAGGTTGTA |  |  |
| LCP17-like-qF | GCTCCCCCAGTCATTCAGAT | 93 | 96.5 |
| LCP17-like-qR | GGCGATTCCGTTCTCAGTCT |  |  |
| AMP8.1-qF | CCTATGTCGCTGACGAGAAC | 95 | 97.1 |
| AMP8.1-qR | GATGGCAAGGAGCTCAACTA |  |  |
| CP8.50-qF | GACGACAACACCTACACTGGT | 91 | 96.1 |
| CP8.50-qR | GGTATCCACGCTTGTCAGCA |  |  |
| CP15.0-qF | ATGAGGGCTCTGGTTGTCTTG | 112 | 94.9 |
| CP15.0-qR | CCTGGTAGGCTGCGAAGAAC |  |  |
| ACP20-qF | TTCAGCCAGAGGGAGGAGTC | 116 | 106.7 |
| ACP20-qR | CGGTAACCCCCGTAGTTGTC |  |  |
| Pro-resilin-qF | AGCACGTCACCTACCAAGTG | 103 | 94.8 |
| Pro-resilin-qR | CCGTATTCCCTCGATTCCTGA |  |  |
| TUBA1-qF | TTCCTCATCTTCCACTCCTTTG | 99 | 100.8 |
| TUBA1-qR | CAACTTGCTCTTCTTGCCATAAT |  |  |
| TUBB1-qF | CAGAGCTCACGCAACAAATG | 121 | 92.6 |
| TUBB1-qR | TCAACCTCCTTCATGGACATAC |  |  |

Additional table 3. Abundance of genes in transcript per million (TPM) involved in Wnt signaling pathway and cell cycle in the transcriptomes of *C. sapidus* ovigerous setae. Transcriptomes: prepuberty females at early premolt (OE) and late premolt (OL); and spawned adult females (AO). |log2 (fold change)| < 1 is noted by ‘-’ symbols.

| **Seq ID** | **Gene Name** | **Gene** | **Matched NCBI** | **Matched** | **TPM Value** | | | **Log2 (fold change) Value** | | |
| --- | --- | --- | --- | --- | --- | --- | --- | --- | --- | --- |
|  |  | **Abbreviation** | **Accession NO.** | **KO NO.** | **OE** | **OL** | **AO** | **OE vs. OL** | **OL vs. AO** | **OE vs. AO** |
| **Wnt Signaling Pathway** |  |  |  |  |  |  |  |  |  |  |
| TRINITY_DN24931_c0_g1 | Division abnormally delayed protein-like | Dally | XP_050694235.1 | K02306 | 1.63 | 3.6 | 4.43 | 1.14 | - | 1.44 |
| TRINITY_DN8542_c0_g1 | Secreted frizzled-related protein 5-like isoform X1 | FRP | XP_045126696.1 | K02222 | 3.6 | 1.8 | 1.85 | - | - | - |
| TRINITY_DN13300_c0_g1 | Protein-serine O-palmitoleoyl transferase porcupine-like X1 | Porc | XP_045111689.1 | K00181 | 1.32 | 0.35 | 0.64 | -1.92 | - | - |
| TRINITY_DN293_c3_g1 | Casein kinase II subunit beta isoform X1 | CK2β | XP_045109910.1 | K03115 | 2.46 | 8.62 | 7.54 | 1.81 | - | 1.62 |
| TRINITY_DN86_c16_g1 | Casein kinase I alpha | CK1α | XP_045111004.1 | K08957 | 11.25 | 43.45 | 36.38 | 1.95 | - | 1.69 |
| TRINITY_DN638_c0_g1 | Casein kinase II subunit alpha isoform X1 | CK2α | XP_045102795.1 | K03097 | 7.61 | 17.18 | 11.44 | 1.17 | - | - |
| TRINITY_DN2531_c0_g1 | Beta-catenin | β-catenin | ALK24421.1 | K02105 | 5.94 | 21.74 | 18.77 | 1.87 | - | 1.66 |
| TRINITY_DN1049_c0_g1 | Casein kinase I-like isoform X2 | CK1 | XP_037798673.1 | K08960 | 7.58 | 21.04 | 16.80 | 1.47 | - | 1.15 |
| TRINITY_DN12371_c0_g1 | Frizzled-2-like | Frizzled | XP_045132948.1 | K02432 | 2.92 | 4.04 | 4.01 | - | - | - |
| TRINITY_DN28965_c0_g1 | Low-density lipoprotein receptor-related protein 6-like | LRP5/6 | XP_045119694.1 | K03068 | 0.38 | 1.33 | 0.71 | 1.81 | - | - |
| TRINITY_DN24956_c0_g2 | Segment polarity protein dishevelled homolog DVL-3-like X1 | DVL | XP_045121633.1 | K02353 | 1.45 | 2.74 | 2.25 | - | - | - |
| TRINITY_DN2623_c2_g1 | Axin-1-like isoform X1 | AXIN1 | XP_045101225.1 | K02157 | 0.98 | 2.12 | 1.90 | 1.10 | - | - |
| TRINITY_DN67718_c0_g1 | Protein pangolin, A/H/I/S-like isoform X6 | TCF | XP_045129768.1 | K04491 | 0.35 | 0.29 | 0.11 | - | -1.40 | -1.67 |
| TRINITY_DN3910_c1_g2 | Protein groucho-like isoform X5 | GRO | XP_042872057.1 | K04497 | 0.82 | 4.14 | 2.18 | 2.34 | - | 1.41 |
| TRINITY_DN3698_c1_g1 | Glycogen synthase kinase-3 beta-like isoform X6 | GSK3β | XP_045126539.1 | K03083 | 0.37 | 1.78 | 1.47 | 2.27 | - | 1.99 |
| TRINITY_DN6120_c0_g1 | RuvB-like 1 | Pontin52 | XP_045101606.1 | K04499 | 2.56 | 3.03 | 5.81 | - | - | 1.18 |
| TRINITY_DN564_c0_g1 | C-terminal-binding protein-like isoform X4 | CtBP | XP_050690287.1 | K04496 | 2.79 | 4.74 | 5.24 | - | - | - |
| TRINITY_DN15149_c0_g1 | G1/S-specific cyclin-D2-like | cycD | XP_045103140.1 | K10151 | 8.03 | 2.75 | 0.18 | -1.55 | -3.93 | -5.48 |
| TRINITY_DN1489_c0_g1 | Transcription factor AP-1 | c-jun | MPC75023.1 | K04448 | 4.98 | 7.89 | 13.47 | - | - | 1.44 |
| TRINITY_DN19894_c0_g2 | N-myc proto-oncogene protein-like | c-myc | XP_045106931.1 | K04377 | 2.86 | 0.93 | 0.91 | -1.62 | - | -1.65 |
| TRINITY_DN24127_c0_g1 | Protein naked cuticle homolog 1-like | NKD | XP_045105601.1 | K03213 | 0.01 | 0.47 | 0.35 | 5.55 | - | 5.13 |
| TRINITY_DN4748_c0_g1 | Ring-box protein 1A | RBX1 | XP_050735003.1 | K03868 | 26.72 | 51.05 | 51.27 | - | - | - |
| TRINITY_DN75402_c0_g1 | S-phase kinase-associated protein 1 isoform X1 | SKP1 | XP_045138954.1 | K03094 | 9.96 | 20.11 | 20.42 | - | - | - |
| TRINITY_DN7116_c0_g1 | Calcyclin-binding protein-like | SIP | XP_045123158.1 | K04507 | 6.06 | 9.64 | 12.21 | - | - | - |
| TRINITY_DN4213_c0_g1 | Cullin-1-like | CUL1 | XP_045124924.1 | K03347 | 4.48 | 6.63 | 8.69 | - | - | - |
| TRINITY_DN7563_c1_g1 | F-box-like/WD repeat-containing protein TBL1XR1 isoform X1 | TBL1 | XP_045106087.1 | K04508 | 2.22 | 2.19 | 2.93 | - | - | - |
| TRINITY_DN1898_c0_g1 | E3 ubiquitin-protein ligase Siah1 | Siah1 | MPC10368.1 | K04506 | 1.05 | 4.49 | 6.91 | 2.10 | - | 2.72 |
| TRINITY_DN9624_c4_g1 | Beta-TrCP-like isoform X1 | β-TrCP | XP_045135295.1 | K03362 | 0.41 | 1.77 | 1.74 | 2.11 | - | 2.09 |
| TRINITY_DN8732_c0_g1 | Inversin-like isoform X1 | INVS | XP_045115266.1 | K19626 | 1.31 | 1.6 | 1.09 | - | - | - |
| TRINITY_DN6096_c2_g1 | Glypican-4-like isoform X1 | Knypek | XP_045113684.1 | K08110 | 0.99 | 4.25 | 3.27 | 2.10 | - | 1.72 |
| TRINITY_DN522_c1_g1 | Vang-like protein 1 | Stbm | XP_045130015.1 | K04510 | 0.68 | 1.5 | 3.07 | 1.14 | - | 2.17 |
| TRINITY_DN8164_c7_g1 | Protein prickle-like isoform X1 | PRICKLE | XP_045135159.1 | K04511 | 0.46 | 1.03 | 0.17 | -1.16 | -2.60 | -1.44 |
| TRINITY_DN1686_c1_g1 | Serine/threonine-protein phosphatase 2B catalytic subunit 3-like isoform X1 | CaN | XP_045102236.1 | K04348 | 4.61 | 14.63 | 14.84 | 1.67 | - | 1.69 |
| TRINITY_DN270_c1_g1 | Protein kinase C, brain isozyme-like | PKC | XP_045123934.1 | K02677 | 3.2 | 11.21 | 5.22 | 1.81 | -1.10 | - |
| TRINITY_DN208_c0_g2 | Calcium/calmodulin-dependent protein kinase type II alpha chain-like isoform X10 | CAMK2 | XP_045104274.1 | K04515 | 2.46 | 4.95 | 6.41 | - | - | 1.38 |
| TRINITY_DN3708_c0_g1 | 1-phosphatidylinositol 4,5-bisphosphate phosphodiesterase classes I and II-like isoform X6 | PLC | XP_045109783.1 | K05858 | 0.65 | 1.35 | 5.45 | - | 2.01 | 3.07 |
| TRINITY_DN378_c14_g1 | Presenilin-2-like | PS-1 | XP_042238824.1 | K04505 | 0.56 | 2.99 | 13.31 | 2.42 | 2.15 | 4.57 |
| TRINITY_DN2030_c4_g1 | Mitogen-activated protein kinase kinase kinase 7-like X3 | Tak1 | XP_045110912.1 | K04427 | 0.65 | 2.57 | 1.14 | 1.98 | -1.17 | - |
| TRINITY_DN1813_c0_g1 | cAMP-dependent protein kinase catalytic subunit 1 isoform X3 | PKA | XP_045117222.1 | K04345 | 1.39 | 5.19 | 7.06 | 1.90 | - | 2.34 |
| TRINITY_DN17155_c0_g1 | Serine/threonine-protein kinase NLK2-like | NLK | XP_045132759.1 | K04468 | 1.44 | 4.72 | 1.08 | 1.71 | -2.13 | - |
| TRINITY_DN969_c1_g1 | Mothers against decapentaplegic homolog 4-like | SMAD4 | XP_045105656.1 | K04501 | 1.37 | 3.27 | 27.8 | 1.26 | 3.09 | 4.34 |
| TRINITY_DN5394_c1_g1 | CREB-binding protein-like isoform X1 | CBP | XP_045101543.1 | K04498 | 1.35 | 3.48 | 4.14 | 1.37 | - | 1.62 |
| TRINITY_DN18576_c0_g3 | Protein Wnt-2b-A-like isoform X1 | WNT2 | XP_045118046.1 | K00182 | 0.11 | 0.57 | 0.05 | 2.37 | -3.51 | -1.14 |
| TRINITY_DN12886_c0_g1 | Wnt-5b-like | WNT5 | XP_045131549.1 | K00444 | 0.15 | 1.52 | 0.23 | 3.34 | -2.72 | - |
| TRINITY_DN47827_c0_g1 | Protein Wnt-6-like isoform X3 | WNT6 | XP_045122246.1 | K00445 | 0.06 | 0.58 | 0.46 | 3.27 | - | 2.94 |
| TRINITY_DN18576_c0_g2 | Wnt-7b-like isoform X1 | WNT7 | XP_045111453.1 | K00572 | 1.12 | 1.22 | 0.17 | - | -2.84 | -2.72 |
| TRINITY_DN15180_c0_g1 | Wnt-11b-like isoform X1 | WNT11 | XP_045120715.1 | K01384 | 0.18 | 0.36 | 0.29 | - | - | - |
| TRINITY_DN95383_c0_g1 | Protein Wnt-16 | WNT16 | MPC34974.1 | K01558 | 0.17 | 0.34 | 0.28 | - | - | - |
| TRINITY_DN9381_c2_g1 | Disheveled-associated activator of morphogenesis 2-like | DAAM | XP_045104670.1 | K04512 | 0.03 | 0.99 | 0.40 | 5.04 | -1.31 | 3.74 |
| TRINITY_DN348_c0_g1 | Ras-like GTP-binding protein RHO isoform X1 | RHOA | XP_045101211.1 | K04513 | 71.17 | 182.84 | 174.52 | 1.36 | - | 1.29 |
| TRINITY_DN2607_c1_g1 | Rho-associated protein kinase 2-like | ROCK2 | XP_045134142.1 | K17388 | 1.72 | 3.26 | 4.24 | - | - | 1.30 |
| TRINITY_DN1689_c0_g1 | Ras-related protein Rac1 isoform X2 | RAC1 | XP_045129822.1 | K04392 | 10.17 | 15.46 | 23.80 | - | - | 1.23 |
| TRINITY_DN7434_c0_g1 | C-Jun N-terminal kinase | JNK | QDF82318.1 | K04440 | 1.96 | 3.77 | 5.41 | - | - | 1.46 |
| **Cell Cycle** |  |  |  |  |  |  |  |  |  |  |
| TRINITY_DN6924_c1_g1 | Cyclin-dependent kinase 2-like | CDK2 | XP_045105810.1 | K02206 | 4.60 | 5.77 | 4.27 | - | - | - |
| TRINITY_DN15149_c0_g1 | G1/S-specific cyclin-D2-like | CycD2 | XP_045103140.1 | K10151 | 2.75 | 8.03 | 0.18 | 1.55 | -5.48 | -3.93 |
| TRINITY_DN4748_c0_g1 | Ring-box protein 1A | RBX1 | XP_050735003.1 | K03868 | 26.72 | 51.05 | 51.27 | - | - | - |
| TRINITY_DN75402_c0_g1 | S-phase kinase-associated protein 1 isoform X1 | SKP1 | XP_045138954.1 | K03094 | 9.96 | 20.11 | 20.42 | - | - | - |
| TRINITY_DN4213_c0_g1 | Cullin-1-like | CUL1 | XP_045124924.1 | K03347 | 4.48 | 6.63 | 8.69 | - | - | - |
| TRINITY_DN12728_c0_g1 | S-phase kinase-associated protein 2 | SKP2 | MPC17822.1 | K03875 | 0.79 | 4.03 | 0.99 | 2.35 | -2.03 | - |
| TRINITY_DN12528_c0_g2 | Cyclin-dependent kinase inhibitor 1B-like | Kip1,2 | XP_045104994.1 | K06624 | 0.08 | 0.38 | 0.44 | 2.25 | - | 2.46 |
| TRINITY_DN1879_c10_g1 | Transcription factor E2F4-like | E2F4,5 | XP_045127786.1 | K04682 | 0.99 | 0.91 | 1.14 | - | - | - |
| TRINITY_DN20010_c0_g1 | Retinoblastoma-like protein 1 isoform X1 | RBL1 | XP_045109252.1 | K04681 | 0.58 | 0.91 | 1.31 | - | - | 1.18 |
| TRINITY_DN668_c2_g1 | Proliferating cell nuclear antigen | PCNA | KAG0712274.1 | K04802 | 5.27 | 4.64 | 11.75 | - | 1.34 | 1.16 |
| TRINITY_DN9609_c0_g1 | Cyclin A | CycA | ADK13092.1 | K06627 | 0.23 | 0.24 | 2.79 | - | 3.54 | 3.60 |
| TRINITY_DN2362_c0_g1 | Cell division control protein 45 homolog | CDC45 | XP_045106468.1 | K06628 | 47.71 | 50.93 | 140.57 | - | 1.46 | 1.56 |
| TRINITY_DN5974_c0_g2 | Cyclin-dependent kinase 7-like | CDK7 | XP_045138847.1 | K02202 | 0.79 | 1.63 | 1.60 | - | - | - |
| TRINITY_DN1669_c1_g1 | Cyclin H | CycH | ACL81559.1 | K06634 | 2.47 | 5.20 | 4.15 | - | - | - |
| TRINITY_DN18538_c0_g1 | Origin recognition complex subunit 2-like | ORC2 | XP_045137366.1 | K02604 | 0.54 | 0.87 | 0.76 | - | - | - |
| TRINITY_DN51933_c0_g1 | Origin recognition complex subunit 3-like | ORC3 | MPC07523.1 | K02605 | 0.32 | 0.39 | 0.19 | - | - | - |
| TRINITY_DN6926_c1_g1 | Cell division cycle 7-related protein kinase-like | CDC7 | XP_045126775.1 | K02214 | 0.51 | 1.26 | 0.77 | 1.30 | - | - |
| TRINITY_DN4971_c0_g1 | Serine/threonine-protein kinase Chk2 | CHEK2 | MPC11584.1 | K06641 | 1.32 | 3.1 | 1.65 | 1.23 | - | - |
| TRINITY_DN51183_c0_g2 | Serine-protein kinase ATM-like | ATM | XP_045119407.1 | K04728 | 2.38 | 3.07 | 2.63 | - | - | - |
| TRINITY_DN5677_c0_g1 | Serine/threonine-protein kinase ATR-like | ATR | XP_045137867.1 | K06640 | 0.50 | 0.68 | 0.95 | - | - | - |
| TRINITY_DN5672_c6_g1 | Cyclin B | CycB | QPO25105.1 | K05868 | 0.64 | 0.30 | 2.56 | -1.10 | 3.09 | 2.00 |
| TRINITY_DN2659_c0_g1 | Cyclin-dependent kinase 1-like | CDK1 | XP_045112582.1 | K02087 | 1.19 | 0.51 | 3.85 | -1.22 | 2.92 | 1.69 |
| TRINITY_DN1737_c9_g1 | Anaphase-promoting complex subunit 13-like | APC13 | XP_045131457.1 | K12456 | 16.22 | 21.84 | 21.95 | - | - | - |
| TRINITY_DN24059_c0_g1 | Cell division cycle protein 20 homolog | CDC20 | XP_045123993.1 | K03363 | 0.29 | 0.07 | 0.6 | -2.05 | 3.10 | - |
| TRINITY_DN9541_c0_g1 | Mitotic spindle assembly checkpoint protein MAD2B-like X2 | MAD2L2 | XP_045127902.1 | K13728 | 0.33 | 1.32 | 0.75 | 2.00 | - | 1.18 |
| TRINITY_DN6891_c0_g1 | Dual specificity protein phosphatase CDC14A-like isoform X5 | CDC14 | XP_045134222.1 | K06639 | 1.53 | 4.8 | 6.78 | 1.65 | - | 2.15 |
| TRINITY_DN969_c1_g1 | Mothers against decapentaplegic homolog 4-like | SMAD4 | XP_045105656.1 | K04501 | 1.37 | 3.27 | 27.8 | 1.26 | 3.09 | 4.34 |
| TRINITY_DN5394_c1_g1 | CREB-binding protein-like isoform X1 | P300 | XP_045101543.1 | K04498 | 1.35 | 3.48 | 4.14 | 1.37 | - | 1.62 |
| TRINITY_DN3698_c1_g1 | Glycogen synthase kinase-3 beta-like isoform X6 | GSK3β | XP_045126539.1 | K03083 | 0.37 | 1.78 | 1.47 | 2.27 | - | 1.99 |
| TRINITY_DN19894_c0_g2 | N-myc proto-oncogene protein-like | c-myc | XP_045106931.1 | K04377 | 2.86 | 0.93 | 0.91 | -1.62 | - | -1.65 |
| TRINITY_DN25_c5_g1 | 14-3-3 protein epsilon isoform X2 | 14-3-3 | XP_050711959.1 | K06630 | 33.61 | 55.02 | 40.92 | - | - | - |
| TRINITY_DN1664_c0_g1 | Mothers against decapentaplegic homolog 3-like isoform X1 | SMAD3 | XP_045123030.1 | K04500 | 13.19 | 14.32 | 13.61 | - | - | - |
| TRINITY_DN4146_c0_g1 | Histone deacetylase 1-like | HDAC | XP_045107406.1 | K06067 | 4.59 | 5.56 | 6.51 | - | - | - |
| TRINITY_DN1851_c0_g2 | Mitotic checkpoint protein BUB3-like isoform X1 | BUB3 | XP_045124808.1 | K02180 | 3.27 | 4.3 | 4.75 | - | - | - |
| TRINITY_DN5092_c0_g1 | DNA-dependent protein kinase catalytic subunit-like | DNA-PK | XP_045103217.1 | K06642 | 1.73 | 4.33 | 10.77 | 1.32 | 1.31 | 2.64 |
| TRINITY_DN3190_c1_g1 | serine/threonine-protein kinase PLK1-like | PLK1 | XP_045137394.1 | K06631 | 0.78 | 2.72 | 6.22 | 1.80 | 1.19 | 3.00 |
| TRINITY_DN143_c13_g1 | Mitotic checkpoint serine/threonine-protein kinase BUB1 | BUB1 | MPC84505.1 | K02178 | 0.06 | 3.04 | 2.46 | 5.66 | - | - |
| TRINITY_DN56990_c1_g1 | dual specificity protein kinase TTK-like | MPS1 | XP_045107535.1 | K08866 | 0.01 | 0.17 | 0.49 | 4.09 | 1.53 | 5.36 |
| TRINITY_DN58458_c0_g1 | structural maintenance of chromosomes protein 1A-like | SMC1 | XP_045126349.1 | K06636 | 0.01 | 1.21 | 0.59 | 6.92 | - | 5.88 |
| TRINITY_DN91877_c0_g1 | 14-3-3 protein eta | 14-3-3α | ETO05653.1 | K06644 | 0.01 | 0.01 | 1.39 | - | 7.12 | 7.12 |
