# Supplementary material for: Identifying the genes involved in the egg-carrying ovigerous hair development of the female blue crab Callinectes sapidus: transcriptomic and genomic expression analyses
Source: BMC Genomics. 2023 Dec 11;24:764. doi: 10.1186/s12864-023-09862-9 (PMC10712104; doi:10.1186/s12864-023-09862-9)
Supplement: Supplementary file 4 — Supplementary Material 4 [file 12864_2023_9862_MOESM4_ESM.docx]

List of Additional files

**Additional files**

Additional excel.file 1. Copy numbers of pro-resilin genes among several representative decapod species.

Additional file 1. Comparison of differentially expressed genes (DEGs). Volcano plot of DEGs in the (A) OE vs. OL, (B) OL vs. AO, and (C) OE vs. AO comparisons, and (D) Venn diagram of overlapping DEGs among these comparisons. False Discovery Rate (FDR) is used to find the *P*-value threshold. Abbreviation: Abbreviation: ovigerous setae in the prepuberty females at early (OE) and late premolt (OL) and adult (AO) stages; Up, up-regulation, Down, down-regulation; and ND, no difference. Statistical analysis of the DEGs between the OE and OL libraries included 6,547 genes, 3,764 of which were up-regulated and 2,783 were down-regulated (Additional file 1A). Overall, 7,793 DEGs are found in the OL vs. AO comparison, including 3,712 up-regulated genes and 4,081 down-regulated genes (Additional file 1B). In total, 7,481 DEGs were screened between the OE and AO libraries, with 4,189 up-regulated and 3,292 down-regulated significantly (Additional file 1C). A Venn diagram revealed the shared and unique DEGs among the OE vs. OL, OL vs. AO, and OE vs. AO comparisons (Additional file 1D).

Additional file 2. (A) The number of enriched Kyoto Encyclopedia of Genes and Genomes (KEGG) pathways in the different groups (p < 0.05). (B) Distribution of Gene Ontology (GO) terms assigned to biological process (BP), cellular component (CC) and molecular function (MF). (C) Venn diagram of overlapping KEGG pathways in the different groups. (D) Venn diagram of overlapping GO terms in the different groups. Abbreviation: ovigerous setae in the prepuberty females at early (OE) and late premolt (OL) and adult (AO) stages. The three comparisons shared 958 DEGs, while OE vs. OL, OL vs. AO, and OE vs. AO possessed 780, 1,813, and 1,486 unique DEGs, respectively. The DEGs in the OE vs. OL, OL vs. AO, and OE vs. AO comparisons were significantly enriched in 197, 190, and 184 KEGG pathways, respectively (Additional file 2A-B). A total of 395, 320, and 455 GO terms were enriched in the OE vs. OL, OL vs. AO, and OE vs. AO comparisons, respectively (Additional file 2C-D). The Kanehisa laboratory have kindly provided permission ([www.kegg.jp/kegg/kegg1.html](http://www.kegg.jp/kegg/kegg1.html)).

Additional file 3. Phylogenetic analyses of *Wnt5b* (A), *β-catenin* (B), *cyclin D2* (C), *cyclin A* (D), *cyclin H* (E), *CDC20* (F), *cuticle* (G) and *tubulin* (H) genes. The phylogenetic tree was constructed based on the deduced full-length amino acid sequence alignments by the Neighbor-Joining (NJ) algorithm embedded in the MEGA X. Bootstrap consisted of 1000 replicates. The arrow points to *C. sapidus* protein (A-F). Different sizes of predicted amino acids of cuticle and tubulin genes (G-H) were represented by the bars upon the phylogenetic tree. The black dots represented *Callinectes* sequences. The amino acid sequences were retrieved from the NCBI database and the accession numbers were presented in the Additional table 1.

Additional file 4. Identification of genes assigned to the mTOR signalling pathway in the (A) OE vs. OL comparison, (B) OL vs. AO comparison, and (C) OE vs. AO comparison, respectively. Up-, down-, and non-significantly regulated genes in the *C. sapidus* transcriptome are coloured red, blue, and yellow, respectively. Abbreviation: ovigerous setae in the prepuberty females at early (OE) and late premolt (OL) and adult (AO) stages.

The Kanehisa laboratory have kindly provided permission ([www.kegg.jp/kegg/kegg1.html](http://www.kegg.jp/kegg/kegg1.html)).

Additional file 5. Predicted transcriptional factor-binding sites on the 5’ flanking sequence of two tandemly distributed *pro-resilin* genes in *C. sapidus* (Gene ID: Chr_9_Csap_19436 and 19438). The putative binding sites for ecdysteroid-responsive factors are marked in red frame. Abbreviation: Fushi tarazu factor 1 (*Ftz-f1*), Antennapedia (*Antp*), Retinoid X receptor (*RXR*), estrogen receptor (*ER*), and progesterone receptor (*PR*).

**Additional tables**

Additional table 1. The information of the sequences used in phylogenetic analysis.

Additional table 2. Primers used for RT-qPCR in this study, the size of amplicons and amplification efficiency

Additional table 3. Abundance of genes in transcript per million (TPM) involved in Wnt signaling pathway and cell cycle in the transcriptomes of *C. sapidus* ovigerous setae. Transcriptomes: prepuberty females at early premolt (OE) and late premolt (OL); and spawned adult females (AO). |log2 (fold change)| < 1 is noted by ‘-’ symbols.
